# Boosting Nitrate to Ammonia Electroconversion through Hydrogen Gas Evolution over Cu-foam@mesh Catalysts

**DOI:** 10.1021/acscatal.3c00716

**Published:** 2023-06-05

**Authors:** Yuzhen Wang, Abhijit Dutta, Anna Iarchuk, Changzhe Sun, Soma Vesztergom, Peter Broekmann

**Affiliations:** † Department of Chemistry, Biochemistry and Pharmaceutical Science, University of Bern, Freiestrasse 3, 3012 Bern, Switzerland; ‡ State Key Laboratory of Eco-Hydraulics in Northwest Arid Region of China, Xi’an University of Technology, No.5 South Jinhua Road, Xi’an, Shaanxi 710048, China; § National Centre of Competence in Research (NCCR) Catalysis, University of Bern, Freiestrasse 3, 3012 Bern, Switzerland; ∥ MTA−ELTE Momentum Interfacial Electrochemistry Research Group, Eötvös Loránd University, Pázmány Péter sétány 1/A, 1117 Budapest, Hungary

**Keywords:** ammonia synthesis, nitrate reduction, nitrite reduction, Cu foam catalyst, HER-mediated nitrate convective mass transport, high nitrate-to-ammonia partial current density

## Abstract

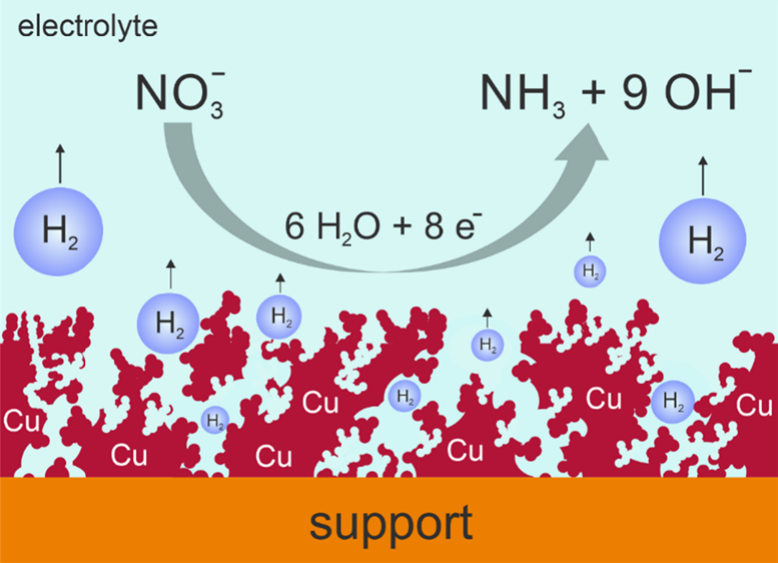

The hydrogen evolution reaction (HER) is often considered parasitic to numerous cathodic electro-transformations of high technological interest, including but not limited to metal plating (e.g., for semiconductor processing), the CO_2_ reduction reaction (CO_2_RR), the dinitrogen → ammonia conversion (N_2_RR), and the nitrate reduction reaction (NO_3_^–^RR). Herein, we introduce a porous Cu foam material electrodeposited onto a mesh support through the dynamic hydrogen bubble template method as an efficient catalyst for electrochemical nitrate → ammonia conversion. To take advantage of the intrinsically high surface area of this spongy foam material, effective mass transport of the nitrate reactants from the bulk electrolyte solution into its three-dimensional porous structure is critical. At high reaction rates, NO_3_^–^RR becomes, however, readily mass transport limited because of the slow nitrate diffusion into the three-dimensional porous catalyst. Herein, we demonstrate that the gas-evolving HER can mitigate the depletion of reactants inside the 3D foam catalyst through opening an additional convective nitrate mass transport pathway provided the NO_3_^–^RR becomes already mass transport limited prior to the HER onset. This pathway is achieved through the formation and release of hydrogen bubbles facilitating electrolyte replenishment inside the foam during water/nitrate co-electrolysis. This HER-mediated transport effect “boosts” the effective limiting current of nitrate reduction, as evidenced by potentiostatic electrolyses combined with an operando video inspection of the Cu-foam@mesh catalysts under operating NO_3_^–^RR conditions. Depending on the solution pH and the nitrate concentration, NO_3_^–^RR partial current densities beyond 1 A cm^–2^ were achieved.

## Introduction

The so-called nitrogen cycle^1−4^ is crucial for the entire global ecosystem. This cycle refers to a circular sequence of geochemical and biochemical transformations through which dinitrogen—an abundant but typically inert source of nitrogen in our atmosphere—is chemically fixed and further transformed into other more reactive forms of “inorganic” nitrogen, e.g., ammonia (NH_3_) or ammonium (NH_4_^+^). This natural nitrogen fixation, the subsequent nitrification, and biochemical assimilation form the basis for the food chain supporting almost all living organisms on Earth. In nature, the nitrogen cycle is closed through the re-ammonification of “organic” nitrogen, e.g., fixed in proteins, through the action of bacteria, and subsequent de-nitrification, which releases the formed dinitrogen back into the atmosphere.^2^ The development of advanced chemical means for nitrogen fixation and its utilization on large industrial scales through the Haber–Bosch process are typically considered as the beginning of the excessive production and use of fertilizers (e.g., ammonium nitrate and ammonium sulfate) and, relatedly, the origin of the unabated growth of the world’s population to date.^5,6^ These unfortunate environmental developments have led to a steady increase in nitrate (NO_3_^–^) concentrations in soil, surface waters, and groundwaters, and alarmingly high nitrate concentrations of 1500 mg L^–1^ have been reported in heavily polluted areas.^7^ This serious environmental threat has high potential to destabilize terrestrial and marine ecosystems, even on a global level.^4−6,8,9^ Extraordinarily high nitrate concentrations in drinking water are also known to severely affect human health, e.g., in the form of methemoglobinemia in infants and gastrointestinal cancer in adults.^10^ Therefore, the World Health Organization recommends to limit nitrate concentrations in drinking water to values below 50 mg L^–1^.^10,11^

To address these environmental problems, various technologies have been developed in the past, on the basis of methods including, but not limited to, ion exchange separation,^12^ reverse osmosis,^13^ or electrodialysis,^14^ mainly with the aim of removing environmentally harmful nitrate from heavily polluted wastewater. In addition, electrolysis techniques have been successfully introduced that allow for the electro-reductive transformation of nitrate into dinitrogen (N_2_), a process often referred to as electrochemical denitrification.^15,16^ More recently, other electrochemical approaches to nitrate reduction have attracted substantial attention because they provide further means of producing nitrogen containing platform chemicals with a higher added value. Potential target molecules of interest are hydroxylamine,^17,18^ a precursor in the production of caprolactam, and ammonia.^18^ The latter is a valuable intermediate in the chemical product chain, e.g., for fertilizer production, and a highly promising carbon-free energy carrier which is, owing to its high energy density of 4.3 kWh kg^–1^, expected to play a central role in the energy transition.^19−21^ If the electrochemical reduction of nitrate (hereafter denoted NO_3_^–^RR) was powered by renewable energy originating from solar radiation, wind power, or hydroelectric sources, this process could become truly sustainable and have high potential to contribute closing not only the nitrogen but also the anthropogenic carbon cycle through the decentralized production of “green” ammonia. Centralized ammonia production via the Haber–Bosch process not only consumes enormous amounts of energy but also substantially contributes to anthropogenic CO_2_ emissions, mainly because the required hydrogen is produced through the environmentally harmful steam reforming of natural gas.^19,22^

In general, a variety of gaseous and liquid products can be yielded from NO_3_^–^RR covering the whole spectrum of possible nitrogen oxidation states (e.g., NO_*x*_, NO_2_^–^, N_2_, hydrazine, hydroxylamine, and ammonia). A comprehensive overview has been provided by Rosca et al.^18^ This diversity of possible reaction products requires the use of electrocatalysts meant not only to accelerate the NO_3_^–^RR but also to steer the reaction selectivity toward the target product. In earlier studies, various transition metals (e.g., Pt,^23−25^ Pd,^26^ Rh,^26^ and Ru^26,27^), coinage metals (Au,^26^ Ag,^26^ and Cu^26,28^), and in some cases oxidic precursors^20^ have been successfully applied as NO_3_^–^RR catalysts. Among the mono-metallic materials studied to date, Ni,^29^ Co,^30^ and Cu^20,26,31,32^ stand out due to their superior NO_3_^–^RR activity and selectivity reaching Faradaic efficiencies (FEs) for ammonia above 90% at comparably low overpotentials (for a survey, see the Supporting Information).

An important aspect of electrocatalyst design concerns the electrochemically active surface area (ECSA), defining the fraction of the catalyst’s surface area accessible to the typically liquid (aqueous) electrolyte in which the reactants are dissolved. Common approaches for maximizing the ECSA often rely on the use of nanoparticulate catalyst materials, which are easily accessible through mature colloid synthesis routes.^33^ An intrinsic drawback of colloidal electrocatalysts is associated with their thermodynamic instability, particularly when their dimensions are within the range of several nanometers. The primary advantage of applying catalysts with a high surface to volume ratio can thus become a disadvantage because this instability can be a source of severe structural alterations during electrolysis, which are often accelerated by the high current densities (reaction rates) required for industrial applications.^34−36^ If material costs are of minor importance, the electrodeposition of metal foams is often considered a promising alternative for fabricating high surface area electrocatalysts. Mono-metallic and alloyed metal foams have already demonstrated superior performance in a variety of electrolysis reactions.^29,37−40^ An important characteristic of metal foams relates to the creation of confined reaction spaces in which the local pH may substantially deviate from the bulk electrolyte under operating conditions, and reaction intermediates can be trapped and be involved in further reaction steps. These “trapping” effects help to mitigate undesired losses of intermediates into the electrolyte, thus often increasing product yields.^38^ However, this apparent advantage of using highly porous catalyst materials can also become a conceptual disadvantage, particularly when high reaction rates are reached, at which the target reaction becomes limited by the reactant mass transport into the three-dimensional structure of the metal foam catalyst.^29^ Related performance losses under such mass transport limitations may become further amplified when the target reaction is superimposed on a parasitic side reaction, which itself does not become mass transport limited. This commonly encountered scenario is relevant to many reductive transformations of technological interest performed in aqueous media, wherein the primary target process often competes with the parasitic hydrogen evolution reaction (HER), thus serving as a source of insufficient FEs of the target reaction.^29^ This is particularly true when higher overpotentials need to be applied in order to achieve reasonable reaction rates at given reactant concentrations as detailed in this work. In the present study, we demonstrate that the gas-evolving HER, e.g., when combined with a porous Cu foam catalyst, can boost the NO_3_^–^RR rate (partial current density, PCD) by opening an additional “convective” reactant transport pathway into the three-dimensional foam structure. Due to their interconnected pore architecture and multi-level porosity,^38,41,42^ Cu foams are ideal model systems to study these transport effects on the performance of such porous high surface area catalyst materials. The formation, growth, and release of hydrogen gas bubbles from the pores of the foamy catalyst mitigate the reactant depletion inside the porous structure through effective electrolyte replenishment. The mechanistic prerequisite for “boosting” the NO_3_^–^RR rate by the HER is a nitrate reduction process that is already diffusion limited. Only under such limiting mass transport conditions can the NO_3_^–^RR rate be influenced by convection mediated either by gas evolution or by other means of electrolyte “stirring”. In this sense, the gas-evolving HER may even become an inherent part of the process and catalyst design, as demonstrated herein.

## Experimental Section

### Catalyst Preparation

Cu foams were electrochemically deposited on a Cu mesh support (0.2 mm thickness, GoodFellow, 99.8% purity) through the dynamic hydrogen bubble template (DHBT) method.^37,42,43^ The nominal aperture and the wire diameter of the mesh support were 380 and 250 mm, respectively. Before electrodeposition, the mesh was cut into pieces 8 mm in width × 25 mm in length. However, a surface area of only 8 mm × 6.25 mm was exposed first to the metal plating bath and later, in the actual electrolysis process, to the nitrate-containing working electrolyte. To ensure that a well-defined geometric surface area was always exposed to the electrolyte, we masked the center parts of the meshes with an insulating Teflon tape (Figure S1). The aqueous plating bath contained 0.2 mol L^–1^ CuSO_4_ as the Cu source (Sigma-Aldrich, ≥98%), which was dissolved in 50 mL of 1.5 mol L^–1^ H_2_SO_4_. For the galvanostatic Cu electrodeposition process, a three-electrode setup was used, with the Cu mesh and a Pt foil serving as the working electrode and the counter electrode, respectively. An Ag/AgCl_3M_ electrode (Metrohm, double junction design) was used as the reference electrode. The experimental setup used for the metal foaming has been detailed in ref (29).

For dedicated control experiments, Cu film catalysts were prepared from the electrodeposited dendritic Cu foams. For this purpose, Cu-foam@mesh samples were transferred after the initial electrodeposition into small vials containing isopropanol and then subjected to 20 min of ultrasonication. As a result, the porous Cu foam was removed from the mesh support and dispersed in isopropanol. Powders of dendritic Cu were obtained through evaporation of the isopropanol solvent at 40 °C for 12 h. For the ink formulation, the dried powders were mixed with isopropanol and a 5 wt % Nafion binder and then subjected to 30 min of sonication. The prepared catalyst ink was drop-cast onto carbon support (gas diffusion electrode type A8, Fuel Cell, USA) with a resulting catalyst Cu mass loading of ∼10 mg cm^–2^, as determined by gravimetry.

For further reference experiments, Cu foams were electrodeposited on a polycrystalline Cu disk (5 mm diameter, Mateck, Germany) following the same protocol as for the Cu mesh supports. Rotating disk electrode (RDE) experiments were carried out using a standard setup from Pine Research (USA).

Cu foam catalysts were also electrodeposited onto planar Cu wafer coupons (Hionix blanket wafer, provided by BASF SE, Ludwigshafen, Germany) that comprised a 100 nm thick PVD Cu seed layer, a 25 nm thick Ta layer, and a 500 nm TO_*X*_/SiO_2_ film on a 5 mm thick Si(100) substrate.

### Elemental Analysis

The post-electrolysis detection and quantification of Cu in the working electrolytes was performed through inductively coupled plasma mass spectrometry (ICP-MS) with a NExION-2000 instrument (Perkin Elmer). For this purpose, aliquots of 20 mm^3^ electrolyte were diluted in 10 cm^3^ of 2 w% HNO_3_ solution (500× dilution). Sample solutions were measured four times each. Repeated measurements served as the basis for the determination of the relative standard deviation. ICP-MS-associated relative standard deviation values are typically between 1 and 2%. An additional measurement error of approximately 1.5% was considered because of the dilution treatment.

### Structural Characterization of the Catalysts

The morphological analysis of the Cu mesh, Cu foam, and Cu film samples was performed with a Zeiss Gemini 450 instrument equipped with an InLens/secondary electron and a backscattered electron detector. For the InLens and backscattered electron detection modes, accelerating voltages (electron currents) of 3.0 kV (100 pA) and 20 kV (1.5 nA) were used as standard settings. AZtec 4.2 software (Oxford Instruments) was applied to acquire energy-dispersive X-ray point spectra and the respective 2D elemental mappings. Surface analysis of the blanket wafer coupons (reference samples) was conducted by tapping mode AFM (Nanosurf Easy Scan II). The surface characterization of the Cu foams deposited on a RDE was further carried out using a 3D digital microscope with focus variation (VHX600, Keyence).

### Electrochemical Characterization

All further electrochemical experiments were performed with a divided H-type electrolysis cell in combination with a classical three-electrode configuration (Figure S2), wherein the Cu-foam@mesh samples (Figure S3) served as the working electrodes, a Pt-foil served as the counter electrode, and an Ag/AgCl_3M_ (Pine research, 3.5 mm outer diameter and 74 mm length) served as the reference electrode. The ECSA of the electrodeposited Cu catalysts was determined through cyclic voltammetry with a dimethyl-viologen redox-active probe described elsewhere (see also Figure S4).^38,44,45^ NO_3_^–^RR experiments were performed with a divided H-type electrolysis cell (Figure S2), wherein the catholyte and anolyte compartments were separated by an anion exchange membrane (Sustainion X37-50 RT, Fuel cell) and, for standard catalyst screening experiments, were filled with 15 mL of 1 mol L^–1^ KOH (pH ∼ 14) or 0.5 mol L^–1^ K_2_SO_4_ electrolyte solution (pH ∼ 7) containing 10, 100, or 500 mmol L^–1^ of KNO_3_ (Sigma-Aldrich, ≥99.0%) as the nitrate source. Before electrolysis, the catholyte was purged for 30 min with Ar gas (99.999%, Carbagas, Switzerland) to remove dissolved oxygen and to prevent an oxygen reduction reaction, which is considered a parasitic side-reaction to the NO_3_^–^RR. Ar flow through the headspace of the cathode compartment was continued during the electrolyses. Electrolyses were performed potentiostatically in a range from −0.7 to −1.6 vs Ag/AgCl_3M_. All electrode potentials reported herein were *iR*-corrected [with cell resistance determined with the current interrupt method in Nova software (Autolab)]. For better comparability, all electrode potentials were further converted to the RHE scale according to
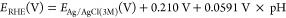
1RDE experiment on Cu foam: A Cu disk electrode (5 mm in diameter, surface area of 0.196 cm^2^) was purchased from Pine Research. The Cu disk electrode was polished using a polishing kit (0.05, 0.3, and 5 μm Alumina suspensions) from PINE Instruments prior to depositing the Cu foam (30 s). Linear sweep voltammetry (LSV, sweep rate 25 mV s^–1^) was performed under stationary conditions applying rotational speeds ranging from 200 to 1600 rpm.

### Product Analysis

Unless otherwise stated, aliquots of the catholyte were collected after 30 min of electrolysis and subjected to quantitative ammonia, nitrite, and nitrate analysis. Ammonia quantification was performed with the standard indophenol blue method (Figure S5 in the Supporting Information).^29^

The FE for ammonia production (FE_NH3_) was derived as the ratio of the charge consumed for NH_3_ formation and the total charge (*Q*_tot_) passed through the cell during the electrolysis according to
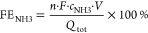
2The total charge *Q*_tot_ (C) passed through the cell during the electrolysis was derived from integration of the respective electrolysis current vs electrolysis time traces. *F* refers to the Faraday constant (96,485 C mol^–1^), *c*_NH_3__ (mol L^–1^) denotes the spectroscopically determined NH_3_ concentration, *V* (L) represents the volume of the catholyte (*V* = 0.015 L), and *n* is the number of transferred electrons.

The quantification of NO_3_^–^ and NO_2_^–^ (the latter of which is a possible byproduct of NO_3_^–^RR) was based on ion-exchange chromatography measurements performed with a Metrohm 940 Professional IC Vario instrument operated with MagIC Net 3.3 software.^29^ For analysis, aqueous solutions of 3 mmol L^–1^ Na_2_CO_3_ and 0.1 mol L^–1^ H_2_SO_4_ served as the eluent and the suppressor, respectively. The IC instrument was calibrated by injection of known standard nitrate and nitrite concentrations (Figure S6) in the range of 10–100 ppm, prepared by dilution of a 1000 ppm IC standard solution (Sigma-Aldrich).

FE values for nitrite were derived from the integrated total charges (*Q*_total_) of the electrolysis reaction and the partial charges (*Q_i_*) corresponding to the formation of a specific product:
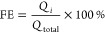
3

The partial charge was calculated according to
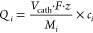
4where *V*_cath_ is the total volume of the catholyte circulating in the electrolyzer, *c_i_* denotes the mass concentration of the product *i* (usually expressed in mg L^–1^ or “ppm”), and *M_i_* is the molar mass of product *i*. After the electrolysis, aliquots of the catholyte solutions were diluted 20- to 100-fold before the IC analysis.

Electrolysis data presented hereafter were acquired by averaging FE and PCD values obtained from three independent electrolysis experiments per applied electrolysis potential.

The so-called nitrogen selectivity (S_*x*_, *x* = NO_2_^–^, NH_3_, others) refers to the ratio of the amount of a certain NO_3_^–^RR product to the total amount of all NO_3_^–^RR products.

## Results and Discussion

**Figure 1:**
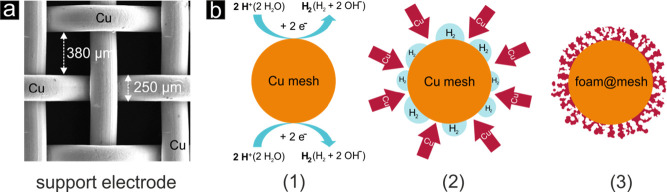
(a) Top-down SEM image of the Cu mesh used in this work as the support electrode for the Cu foam (catalyst) electrodeposition. (b) Cross-sectional schematic illustrating the working principle of the DHBT-assisted Cu foam electrodeposition approach applied to the non-planar mesh support.

Figure 1 depicts the basic principle of the DHBT-assisted electrodeposition of copper,^38,42^ transferred to an open and non-planar mesh type of support material (Figure 1a). The basic concept of this metal-foaming process relies on a gas-evolving process, e.g., the HER, on which the actual metal deposition is superimposed. For this metal-foaming process, extremely harsh experimental conditions must be applied, wherein current densities can exceed several amperes per square centimeter.^37,42,43^ A typical geometric current density often reported in the literature is −3 A cm^–2^.^37,41,44^ Because of substantial proton mass transport limitations at these high rates, the HER is fed by proton reduction not only according to
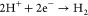
R1but also according to the reductive splitting of water
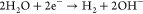
R2

**Figure 2:**
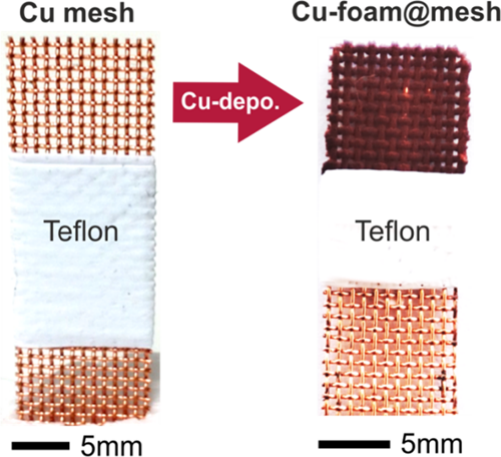
Optical micrographs of the Cu mesh support before and after the Cu foam electrodeposition.

**Figure 3:**
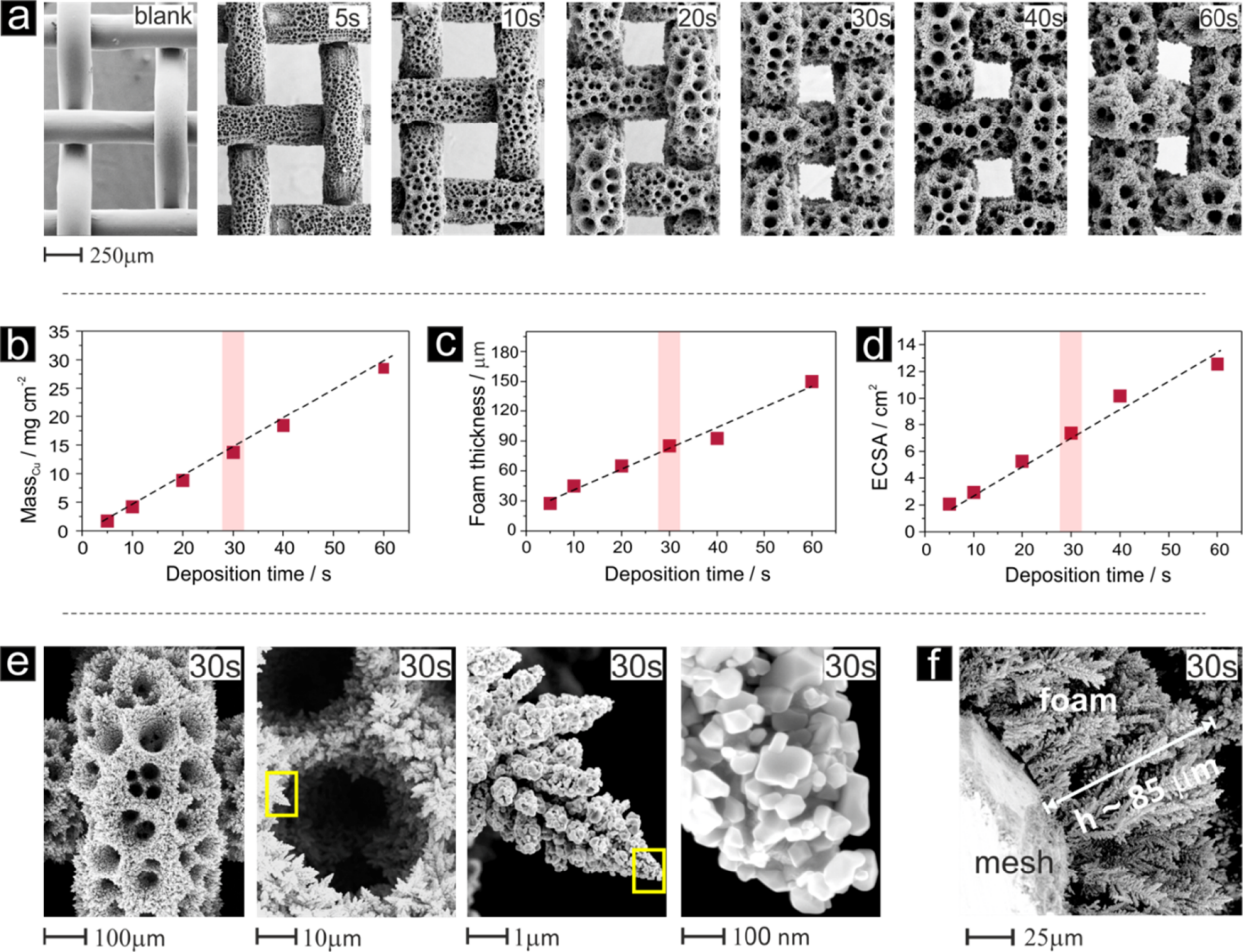
(a) Series of top-down SEM images illustrating the evolution of the foam morphology on the mesh support as a function of the Cu deposition time at a constant geometric current density of −3 A cm^–2^. (b–d) Graphs illustrating the linear dependence of the deposited Cu mass (b), the foam thickness (c), and the electrochemically active surface area (d) on the Cu deposition time. (e) Series of SEM images of increasing magnification showing the mesoscopic and nanoscopic morphology of a (f) representative Cu foam exemplified for the 30s deposition time (denoted Cu-foam(30 s)@mesh).

Hydrogen bubbles, which form and adhere temporarily to the surface of the support electrode during the foaming process, serve as a dynamic template for the metal deposition.^37,46^ Consequently, highly porous metal deposits are created on the substrate and, in the present case, cover concentrically the interconnected wires of the mesh support (Figure 1b). The formed catalyst/support ensemble is hereafter denoted Cu-foam@mesh. Figure 2 depicts optical micrographs of the Cu mesh before and after the electrochemical metal-foaming process, showing a dark reddish appearance of the porous Cu deposit. As demonstrated in Figure 3, the hierarchical porosity of the Cu-foam@mesh samples showed substantial alterations when the applied deposition time was changed (see also Figure S3). The primary porosity of the Cu-foam@mesh was governed by the square-shaped “aperture” of the mesh substrate itself. Its initial opening of ∼380 μm, however, shrinks during the course of the metal-foaming process (Figure 3a). Deposition times exceeding 60 s even led to the complete closure of the mesh openings (not considered for NO_3_RR performance analyses in this study). A *secondary porosity* of the Cu-foam@mesh catalyst resulted from the pore size distribution of the covering Cu foam deposit. As a consequence of hydrogen gas bubble coalescence during the course of the metal-foaming process, the surface pore diameters of the Cu foam typically increase with deposition time, thus introducing a gradient of pore sizes along the surface normal into the system with the largest pores in direct contact with the liquid electrolyte.^38,41,42^ Another contributor to the evolution of this pore size gradient was the continued metal growth inside the foam during the foaming process, which resulted in a shrinkage of the pore sizes near the support surface. Gravimetric analysis of the Cu-foam@mesh samples revealed a linear change in Cu mass loading with deposition time, thus indicating a fairly constant FE for the HER and the metal deposition. This finding was due to the Cu deposition, which readily became mass transport limited at the applied (total) geometric current density of TCD_geo_ = −3 A cm^–2^. Cross-sectional SEM analyses of the Cu foam deposits (see representative example in Figure 3f) also indicated a foam thickness that scaled linearly with the deposition time (Figure 3c).

Herein, the ECSA was determined through the “viologen method” for probing the reduction/oxidation processes of a reversible redox-couple.^29,38,41,45^Figure S4 in the Supporting Information shows the sweep rate-dependent voltammetric responses for a series of Cu-foam@mesh samples including the mesh support itself, which served as an internal reference. Plots of the (reduction) peak currents (*j*_p_) against the square root of the sweep rate (ν^1/2^) rate in all cases indicated a linear relationship, thus demonstrating that the recorded voltammetric data obeyed the Randles–Ševčík equation as a conceptional prerequisite for the ECSA determination through such a Faradaic redox probe. ECSA values increased from ∼1.1 cm^–2^ (mesh blanket) initially to 12.55 cm^–2^ (60 s deposition), which corresponded to a surface area increase greater than one order of magnitude (Figure 3d). A more detailed morphological characterization of the 30 s sample (Figure 3e), in full agreement with previous results with planar electrode supports,^37,38,42^ confirmed the dendritic nature of the Cu deposit constituting the pore side walls of the foam material. Individual dendrites of the metal foam are composed of Cu crystallites terminated by well-defined (100) or (111) facets, as reported by Dutta et al.^38,41^

**Figure 4:**
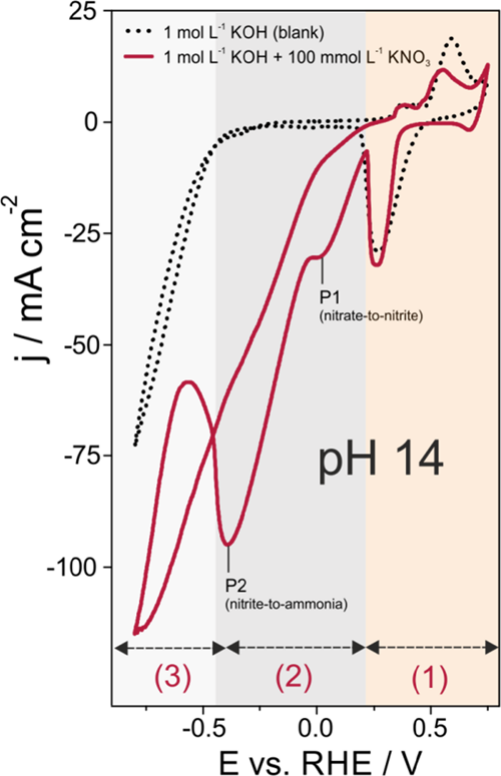
Cyclic voltammograms of a Cu-foam(30 s)@mesh sample recorded in a 1 mol L^–1^ KOH (blank) and 1 mol L^–1^ KOH + 100 mmol L^–1^ KNO_3_ solution. The potential sweep rate was 25 mV s^–1^.

Initial electrochemical characterization of the thus-obtained Cu foam material was performed through cyclic voltammetry in a 1 mol L^–1^ KOH electrolyte solution (blank, pH ∼14) and in the presence of an additional 100 mmol L^–1^ KNO_3_ (Figure 4). Of note, a concentration of 100 mmol L^–1^ KNO_3_ was used for the initial catalyst evaluation to allow for comparison with data in the literature.^29,47−49^ In pH neutral solutions and particularly under alkaline conditions, copper readily undergoes rapid (surface) oxidation after initial O/OH adsorption.^50−55^ The voltammetric responses presented herein were intentionally extended to the anodic potential regime of oxide formation/reduction to reveal the extent to which the targeted NO_3_^–^RR interfered with (i) copper oxidation/reduction processes and (ii) the HER, both of which could be superimposed on the NO_3_^–^RR. As in the case of CO_2_RR,^56,57^ the specific role of (surface) oxides is also under debate for the NO_3_^–^RR.^58,59^ The HER is often considered in the literature to be parasitic to the NO_3_^–^RR, thus decreasing its Faradaic yields.^15^ The accessible potential window in the 100 mmol L^–1^ KNO_3_ solution (pH 14) can be subdivided into three well-separated domains. Pronounced anodic and cathodic features at potentials more positive than ∼+0.2 V vs RHE [denoted (1) in Figure 4] relate to the Cu_*x*_O passive film formation in the positive potential sweep and its electro-reduction into metallic Cu in the corresponding reverse potential sweep.^50−52^ Anodic Cu dissolution into the electrolyte solution after passive film breakdown and cathodic Cu re-deposition in the reverse potential sweep occur in the potential range beyond +0.5 V vs RHE. To avoid structural disintegration of the catalyst foam material, this potential regime was omitted for the catalyst performance testing presented herein. Of note, the surface areas under the oxidation and reduction peaks are slightly different for the blank (1 mol L^–1^ KOH solution; measured first) and the nitrate-containing electrolyte (measured afterward) because the ECSA typically changes during repetitive Cu dissolution/re-deposition processes. Comparison of voltammograms recorded in the nitrate-containing electrolyte and the blanket electrolyte suggested substantial NO_3_^–^RR starting at potentials smaller than ∼ +0.2 V vs RHE. Of note, the width of the cathodic peak associated with the Cu_*x*_O passive film depends on the anodic vertex potential (Figure S7).

The apparent NO_3_^–^RR onset potential was more negative than the stability regime of the Cu_*x*_O passive film, thus demonstrating that nitrate reduction occurred during extended potentiostatic electrolyses predominantly on the metallic Cu without any substantial involvement of (surface) oxide species. The same conclusion was drawn for pH 7 (Figure S8). Cuprous or cupric NO_3_^–^RR catalyst materials or composites thereof reported in the literature^20,60^ are therefore expected to undergo rapid electroreduction at potentials relevant to nitrate reduction and consequently must be considered oxide-derived metallic Cu catalysts.

In the potential regime from approximately +0.2 to −0.45 V vs RHE, two reductive current waves were observed and assigned to the NO_3_^–^RR, which occurred in this potential regime without any interference with the HER [see (2) in Figure 4]. The smaller current feature centered at +0.03 V vs RHE, denoted P1 in Figure 4, corresponds to the conversion of nitrate (oxidation state +5) to nitrite (oxidation state +3) according to R3:
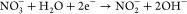
R3

This assignment is in full agreement with the work by Bouzek et al. examining nitrate reduction in (weakly) alkaline solutions through voltammetry in a rotating ring disk electrode (RRDE) configuration.^61^ This early work has also postulated the ready release of NO_2_^–^ from the polycrystalline Cu catalyst into the electrolyte.^61^

We assigned the main cathodic wave P2 centered at −0.38 V vs RHE (Figure 4) to the subsequent nitrite → ammonia conversion according to R4:
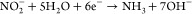
R4

Of note, ammonia can be produced either directly from chemisorbed *NO_2_^–^ intermediates (where * refers to an adsorption state) formed on the catalyst surface in the course of initial nitrate reduction process or through the re-adsorption of afore-released NO_2_^–^ (aq.) species and their further reaction. In the latter scenario, the porous nature of the Cu catalyst enables the trapping of intermediates, thereby facilitating their re-adsorption and further reaction in the interior of the three-dimensional foam material.^37,38^ Additional voltammetric control experiments in an alkaline 100 mmol L^–1^ KNO_2_ solution (Figure S9) showed that nitrite reduction indeed coincided with the main reduction wave P2. All these voltammetric measurements confirmed that, with a nitrate concentration of 100 mmol L^–1^, NO_3_^–^RR (nitrate → ammonia conversion) became mass transport limited at potentials more negative than −0.4 V vs RHE, coinciding with the onset of the HER. The potential domain where NO_3_^–^RR and HER occurred simultaneously is denoted (3) in Figure 4. This conclusion was further confirmed by control experiments in which the Cu-foam(30 s)@mesh catalyst was transferred to a Cu disk electrode support (Figures S10 and S11) and applied to a classical RDE experiment realizing defined stationary transport conditions (Figure S12a,b). The rotation rate depending on linear sweep voltammograms clearly shows the appearance of two (quasi)plateaus associated to mass transport-limited nitrate → nitrite conversion and nitrate/nitrite → ammonia reduction processes.

**Figure 5:**
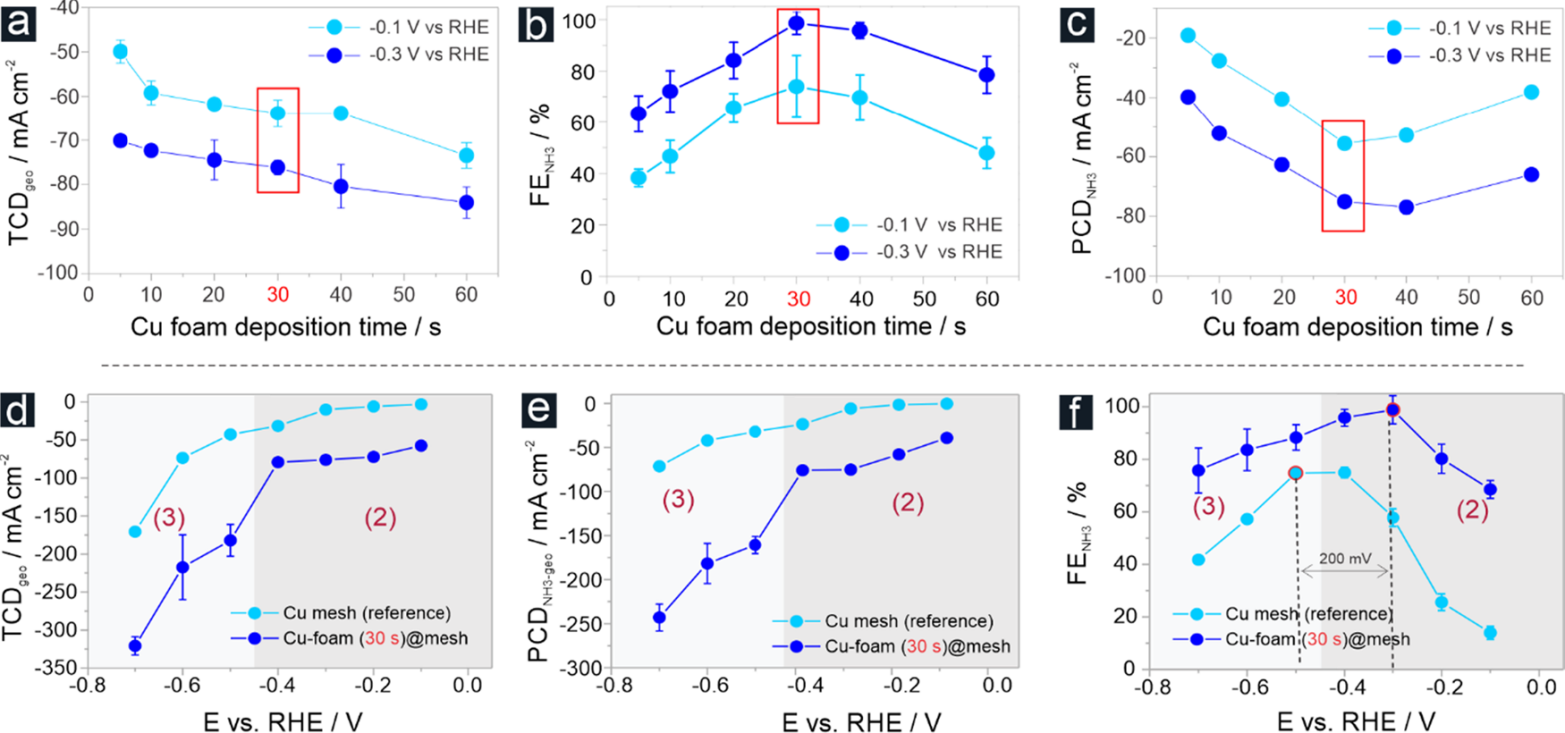
(a) Plot of the total current density (TCD_geo_, normalized to the geometric surface area) as a function of the Cu foam deposition time. The 30 min electrolyses were carried out in a 100 mmol L^–1^ KNO_3_ solution (pH 14) at electrolysis potentials of −0.3 and – 0.7 V vs RHE, respectively. (b) Corresponding plot of the FE for ammonia (FE_NH3_). (c) Corresponding plot of the PCD for ammonia (PCD_NH3_). The Cu-foam(30 s)@mesh sample, identified as the optimum catalyst in this series, is highlighted red in these graphs. (d) Potential-dependent TCD_geo_ values obtained for the Cu-foam(30 s)@mesh catalyst. (e) Corresponding PCD_NH3_ vs E plot. (f) Corresponding FE_NH3_ vs E plot. In (d–f), the three characteristic potential regimes are highlighted according to the voltammogram presented in Figure 4.

**Figure 6:**
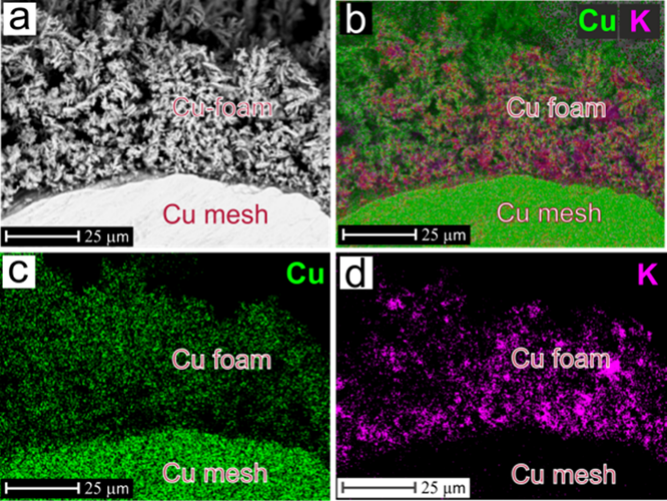
(a) Cross-sectional SEM image of the Cu-foam(30s)@mesh sample (after electrolysis). (b) SEM–EDX-K/Cu mapping, (c) EDX-Cu mapping, and (d) EDX-K mapping.

To probe the effects of the Cu foam morphology (thickness, pore size distribution, etc.) on the electrocatalytic NO_3_^–^RR performance, we performed a set of 30 min potentiostatic electrolyses in 1 mol L^–1^ KOH/100 mmol L^–1^ KNO_3_ electrolyte solutions at dedicated potentials of −0.1 and −0.3 V vs RHE (Figure 5a–c). The Cu-foam@mesh samples (Figure 3a) served as the catalysts. Such short electrolyses were chosen for initial catalyst screening because the total current densities (TCDs)/PCDs and the corresponding FEs are relatively less affected by the nitrate consumption (see the discussion of Figure 8). For catalyst performance evaluation, we therefore restricted ourselves to what we call the “initial” TCD, PCD, and FE values. Representative chrono-amperometric data is provided in Figure S13. Figure 5a indicates an increase in the total current density (TCD_geo_) of electrolysis with Cu deposition time, thus reflecting the observed upward trend of the ECSA with increasing Cu foam thickness (Figure 3b). A post-electrolysis cross-sectional SEM–energy-dispersive X-ray (EDX) analysis of a processed Cu-foam@mesh sample confirmed the complete wetting of the porous foam down to the Cu mesh support (Figure 6). Iarchuk et al. have demonstrated that the spatially resolved elemental mapping of potassium can be used as a chemical fingerprint for the permeation of the electrolyte solution into the three-dimensional structure of the porous foam materials.^29^ Our results demonstrated that not only the PCD for ammonia production (PCD_NH3_) but also the FE_NH3_ changed with the (foam) deposition time (Figure 5b,c), thus indicating that the foam morphology indeed influenced NO_3_^–^RR performance. The Cu-foam(30 s)@mesh sample outperformed in these initial 30 min lasting screening experiments, reaching efficiency values of FE_NH3_ = ∼69% (PCD_NH3_ = ∼−44 mA cm^–2^) and FE_NH3_ = 98.5% (PCD_NH3_ = −75.0 mA cm^–2^) at electrolysis potentials of −0.1 and −0.3 V vs RHE, respectively. The relative performance losses observed for the Cu-foam(60 s)@mesh sample (Figure 5a,b) are likely due to the onset of nitrate mass transport limitations into the considerably thicker Cu foam (see Table S1). Cross-sectional SEM–EDX inspection confirms also in this case the complete wetting of the Cu foam with electrolyte down to the mesh support (Figure S14).

In general, NO_3_^–^RR occurring in the spatially confined volumes of the meso-porous foam structure appeared to be particularly beneficial for the nitrate → ammonia conversion. We have reported similar advantageous morphological effects with a certain optimum in the pore size distribution for other reductive electro-transformations in which either local pH effects play an eminent role in product selectivity, or various intermediates formed in multi-step reactions are trapped and further reacted inside the three-dimensional porous structure. A prime example of the latter is the electrochemical conversion of CO_2_ into hydrocarbons and alcohols.^37,38,41^

The beneficial role of the catalyst morphology for the NO_3_^–^RR was further supported by additional control experiments in which the mesoscopic pore structure of the deposited foam material was intentionally destroyed without affecting the dendritic nature of the sidewalls of the pristine Cu foam. This structure was achieved through ultrasonication of the Cu-foam@mesh catalyst transferring the dendritic Cu deposit from the mesh support into a liquid suspension as a basis for the formulation of a catalyst ink (Figure S15). A Cu film catalyst was yielded by drop-casting this ink onto a carbon support inert with respect to the NO_3_^–^RR. Compared with the best-performing Cu-foam(30 s)@mesh catalyst, this Cu film indeed showed a diminished ammonia FE of only 80% at −0.3 V vs RHE (compared with 98.5% for the Cu-foam(30 s)@mesh, see Figure S16). Of note, even the produced Cu film revealed some nanoscale porosities because of the dendritic nature of Cu.

Based on the above considerations, we concluded that the excellent performance of the Cu-foam (30 s)@mesh originated from an intrinsically high activity of the dendritic Cu (→catalyst morphology on a nm length scale) in combination with additional porosity effects (→catalyst morphology on a μm and mm length scale), e.g., through nitrite trapping (see also discussion in Figure S20).

The Cu-foam(30 s)@mesh, which outperformed in the initial catalyst screening, was further subjected to a comprehensive potential-dependent performance analysis (Figure 5d–f). For reference purposes, the corresponding data of the Cu mesh support is also provided in these graphs. Because of surface area effects, the TCD_geo_ values were generally higher for the Cu-foam(30 s)@mesh catalysts than the mesh reference (Tables S1 and S2). However, the TCD_geo_ values of the Cu-foam(30 s)@mesh catalyst increased only moderately when the applied potential was stepped from −0.1 to −0.2 V vs RHE. A (quasi)plateau in the TCD_geo_ values was observed, ranging from −0.2 to −0.4 V vs RHE (−72.2, −76.2, and −79.2 mA cm^–2^ at −0.2, −0.3, and −0.4 V, respectively; Table S3). However, the electrolysis reaction was substantially accelerated below −0.4 V vs RHE (e.g., TCD_geo_ = −182.0 mA cm^–2^ at −0.5 V vs RHE), and the TCD_geo_ values continued to increase at more cathodic potentials. The corresponding PCDs for ammonia production (PCD_NH3_) followed the same trend, thus indicating a substantial increase in ammonia production rate for potentials below −0.4 V vs RHE (Figure 5e). For example, at an applied potential of −0.7 V vs RHE, the PCD_NH3_ achieved an excellent value of −242.9 mA cm^–2^. From the blank voltammogram recorded in a 1 mol L^–1^ KOH solution (Figure 4), we concluded that the observed sharp increase in both the TCD_geo_ and PCD_geo_ values below −0.4 V vs RHE (Figure 5d,e) coincided with the transition from an electrolysis process governed solely by NO_3_^–^RR to experimental conditions in which nitrate reduction became increasingly superimposed by the gas-evolving HER. These considerations suggested a reactant mass transport predominantly based in potential regime 2 on nitrate diffusion from an unstirred electrolyte solution (Figures 4 and 5e) where (quasi)stationary conditions are reached solely through natural convection.

Initiating the gas-evolving HER below −0.4 V vs RHE led to a change in the hydrodynamic conditions at the catalyst/electrolyte interface and opened an additional convection-assisted nitrate transport pathway. The physical origin of this electrolyte convection was the nucleation, growth, coalescence, and facile release of hydrogen bubbles from the porous foam structure under HER conditions, thus mitigating the depletion of the reactants inside the 3D volume of the foam catalyst material under limiting nitrate transport conditions. Of note, the thickness of the diffusion boundary layer for nitrate anions in quiescent electrolyte solutions (associated with potential regime 2 in Figure 4) extended over time to several hundreds of micrometers, thus not only exceeding the typical pore dimensions of the Cu-foam(30 s)@mesh sample but also surpassing the thickness of the Cu foam covering the mesh support. We hypothesized that under hydrogen gas-evolving conditions, the nitrate diffusion boundary layer thickness substantially decreased, thus boosting the effective limiting PCD of nitrate reduction (PCD_NH3_^limit^). Similar effects have been reported in the literature from electro-foaming processes in which the primary metal deposition is diffusion limited and also superimposed on the HER.^37^ Popov et al. have indicated that these effects can lead to a substantial increase in the limiting current for the metal deposition as a mechanistic prerequisite for the actual metal-foaming process.^46^ For an estimation of the Nernst diffusion layer thicknesses and corresponding achievable limiting currents, we refer to the discussion of Figure S17 in the Supporting Information.

Our working hypothesis of a hydrogen bubble-assisted “boost” of the nitrate reduction rate was further corroborated by a visual inspection of the electrolysis reactions (see the Media file 1 in the Supporting Information). Videos recorded within a broad range of electrolysis potentials indeed confirmed that the gas evolution reaction on the Cu-foam(30 s)@mesh started at −0.4 V vs RHE. The formation and release of hydrogen bubbles became more effective when the applied electrolysis potential was further decreased. However, the clear “boost” in the NO_3_^–^RR rate mediated by the gas-evolving HER came, however, at the expense of certain losses in the FE for ammonia production, as demonstrated in the FE_NH3_ versus E plot (Figure 5f). This also implies a certain losses in the energy efficiency (EE) of the process when compared to conditions where near unity ammonia efficiencies are realized at lower applied overpotentials. A near unity ammonia efficiency of ∼99% was observed for the Cu-foam(30 s)@mesh catalyst only in potential regime 2 (Figures 5f and 4), e.g., at an applied potential of −0.3 V vs RHE (Table S3). The decrease in FE_NH3_ values at potentials more negative than −0.4 V vs RHE therefore was clearly associated with the HER, which began to compete with NO_3_^–^RR for catalytically active surface sites. The production of hydrogen was further confirmed by online gas chromatography (Figure S18).

In this respect, HER can be considered to have a dual role, with (i) a detrimental effect on the FE (and the EE), because of the competition of (adsorbed) hydrogen and NO_3_^–^RR intermediates for catalytically active sites, and (ii) a beneficial effect on the ammonia production rate (PCD), because of accelerated (convective) reactant mass transport and electrolyte replenishment inside the porous catalyst. The accelerated mass transport appears to be a conceptional prerequisite for the benefits of a highly porous three-dimensional catalyst material with an intrinsically high electrochemically active surface area. This aspect is particularly true when the electrocatalytic target reaction (e.g., NO_3_^–^RR) competes with a second electrolysis process that is not affected by any diffusional mass transport limitation (e.g., the reductive water splitting). In the absence of effective reactant transport into the porous catalyst, the high electrochemically active surface area might otherwise become a conceptual disadvantage under such competing reaction conditions.

These statements were further supported by a comparison of the Cu-foam(30 s)@mesh catalyst performance with electrolysis data derived from electrolyses applying ideally planar wafer coupon surfaces as the catalysts (see Figures S19 and S20 and Media file 2). The “boost” of the ammonia PCDs mediated by the HER is much more pronounced in case of the high surface area (3D) foam catalyst than for the ideally planar (2D) wafer coupon catalyst. These supplementary experiments also confirm the effective nitrate trapping at low overpotentials in the presence of a foam structure (see Figure S20c,g in the Supporting Information).

**Figure 7:**
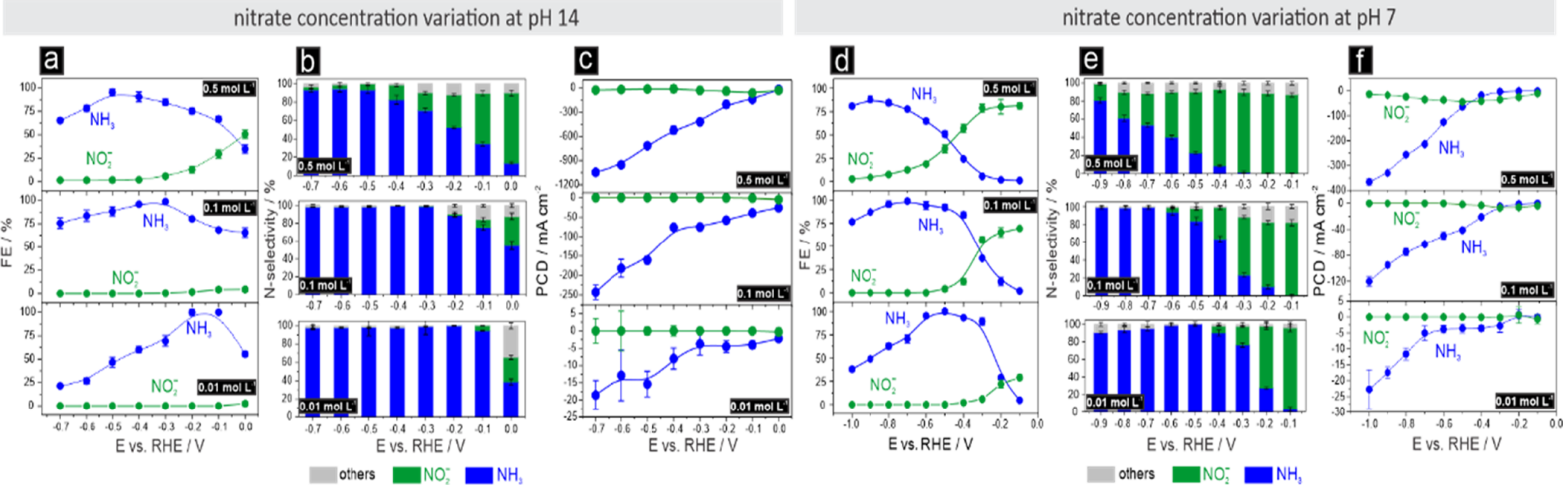
(a) Plots of the potential-dependent FE of ammonia and nitrite production versus the applied electrolysis potential (E vs RHE). For these experiments, the initial nitrate concentration in the electrolyte has been varied (10, 100, and 500 mmol L^–1^) at a fixed pH of 14. All electrolyses were performed for 30 min. (b) Corresponding plots of the potential-dependent nitrogen (N) selectivity for pH 14. (c) Corresponding plots of the potential-dependent PCDs for pH 14. (d) Plots of the FE for ammonia and nitrite vs the applied electrolysis potential (E vs RHE) for electrolyses carried out at pH 7. (e) Corresponding plot of the potential-dependent N-selectivity for pH 7. (f) Potential-dependent PCDs. For all electrolyses, Cu-foam(30 s)@mesh samples were used as the catalysts.

A more comprehensive overview of the product distribution, the N-selectivity (conversion efficiency of nitrate into various N-containing reaction products), and the PCDs for the formation of ammonia and nitrite—the latter of which is the predominant byproduct (and intermediate) of the NO_3_^–^RR with Cu catalysts^61^—is provided in Figure 7. For these additional electrolysis experiments, the initial nitrate concentration and the solution pH were also varied as additional key parameters determining catalyst performance. Results for pH 14 are displayed in Figure 7a–c. Figure 7a demonstrates a near unity FE_NH3_ achieved at pH 14 for all three nitrate concentrations studied herein (500, 100, and 10 mmol L^–1^). However, the maximum ammonia FE shifted toward more negative electrolysis potentials with increasing nitrate concentrations (10 mmol L^–1^: 100% at −0.2 V; 100 mmol L^–1^: 98.5% at −0.3 V; 500 mmol L^–1^: 95.2% at −0.5 V; Table S4). This trend can be explained by two effects. The first relates to the production of nitrite (NO_2_^–^), which can form over Cu as a non-desired product of NO_3_^–^RR which does not further react to ammonia at low overpotentials.^61^ This parasitic reaction becomes increasingly dominant at higher nitrate concentrations (e.g., 500 mmol L^–1^ at pH 14), as demonstrated in Figure 7b, depicting the respective plots of the potential-dependent N-selectivity. At more negative potentials, the nitrate, and presumably also the nitrite → ammonia conversion, becomes accelerated in combination with more effective suppression of the HER, particularly at nitrate concentrations of 100 and 500 mmol L^–1^. This second effect also contributes to the observed cathodic shift in the maximum of the FE_NH3_ values to more cathodic potentials with increasing nitrate concentrations (see Figure 7a).

Figure 7b identifies the low nitrate concentration regime (10 mmol L^–1^) as the optimum at pH 14 regarding the ammonia selectivity (denoted S_NH3_ in Table S5). At 10 mmol L^–1^ nitrate concentration, a near unity S_NH3_ value was achieved within a broad potential regime ranging from −0.1 V to −0.7 vs RHE. However, this potential regime of high ammonia selectivity decreased with increasing nitrate concentrations and, at a nitrate concentration of 500 mmol L^–1^, became limited to the cathodic potential regime below −0.4 V vs RHE. The transition toward an NO_3_^–^RR that was accelerated by the gas-evolving HER was already visible at −0.3 V vs RHE in the respective PCD vs E plot for the 10 mmol L^–1^ case but not for the highest nitrate concentration of 500 mmol L^–1^, simply because the nitrate diffusion limitation had not yet been reached at the onset of the HER in the 500 mmol L^–1^ electrolyte solution (Figure 7c). Additional convection by gas evolution therefore did not have a substantial effect on the resulting PCD_NH3_ values in this case. This conclusion was further confirmed by the RDE experiments presented in Figure S12. The significant increase in the obtained ammonia PCDs (beyond 1 A cm^–2^) with the nitrate solution concentrations should be highlighted (Figure 7c). At an electrolysis potential of −0.7 V vs RHE, the PCD_NH3_ changed from −18.5 mA cm^–2^ to −210.7 mA cm^–2^ to −1045.4 mA cm^–2^ when the nitrate concentration was changed from 10 to 100 mmol L^–1^ and finally 500 mmol L^–1^. Table S16 in the Supporting Information provides a survey of the obtained PCD_NH3_ values reported in the literature in comparison with this work, thus confirming the excellent performance of the Cu-foam(30 s)@mesh catalyst.

Numerous catalyst screening experiments in the literature have been performed under strongly alkaline conditions,^20,27,29,47,49,62,63^ which are, however, of less relevance to many applications. Therefore, we extended the catalyst performance testing to pH 7, which is notably close to that of wastewater treatments.^15^ One clear and important effect of decreasing the solution pH on the NO_3_^–^RR characteristics concerned parasitic nitrite production, which was favored in pH neutral solutions, particularly at elevated nitrate concentrations (Figure 7d,e). Not only were the FE_nitrite_ values and the nitrite selectivity higher at pH 7 (Tables S8–S10) than at pH 14, but the potential window in which the parasitic nitrite byproduct formed was further expanded toward more cathodic potentials when the electrolyses were performed at pH 7 (particularly the 100 and 500 mmol L^–1^ cases in Figure 7d,e). Even at the most cathodic potential of −1.0 V vs RHE applied herein, nitrite appeared as a byproduct when an initial nitrate concentration of 500 mmol L^–1^ was used. The mechanistic origin of the observed trend toward nitrite formation at lower pH is still under debate. However, the involvement of highly active hydrogen species adsorbed on the Cu surface at pH 7 could already be excluded. Recent experimental and theoretical work confirms that highly active copper hydrides are stable only under strong acidic conditions but not at pH 7.^64^

With regard to the NO_3_^–^RR rates, in full agreement with the literature, we observed PCD_NH3_ values generally lower at pH 7 than highly alkaline conditions at pH 14 (Figure 7f). However, the additional “boost” in the PCD_NH3_ values through the gas-evolving HER at pH 7 remained restricted to the 10 mmol L^–1^ nitrate concentration and started at potentials below −0.7 V vs RHE, again in excellent agreement with the voltammetric data (Figure S8). At higher nitrate concentrations of 100 and 500 mmol L^–1^, the increase in PCD_NH3_ was steady with the increasing overpotentials, thus indicating sufficient nitrate mass transport.

The FE and PCD values are important metrics for evaluating catalyst performance; moreover, the long-term stability of the catalyst is important for future industrial applications.^15^ Herein, we conducted dedicated catalyst stability tests in the form of extended potentiostatic electrolyses performed in a discontinuous mode at dedicated potentials of −0.3 and −0.7 V vs RHE. Again, these potentials corresponded to the two characteristic electrolysis potential regimes wherein either solely the NO_3_^–^RR was active or the nitrate reduction became superimposed on the HER. The electrolyses lasted 42 h in total and were subdivided into sequences of 7 h of continuous electrolysis interrupted by electrolyte replenishment steps, which are required when performing extended electrolyses in batch reactors with limited amounts of reactants present. Of note, for such replenishment, the wet Cu-foam(30 s)@mesh catalysts were exposed to air for a certain time which was a source of possible catalyst surface oxidation. In this regard, the catalysts experienced a twofold stress: one due to the electrolysis reaction itself and the other resulting from the repetitive loss of potential control and related surface oxidation/reduction phenomena. According to the voltammetric analysis, formed surface oxides should readily reduce under NO_3_^–^RR conditions (Figures 4 and S8).

**Figure 8:**
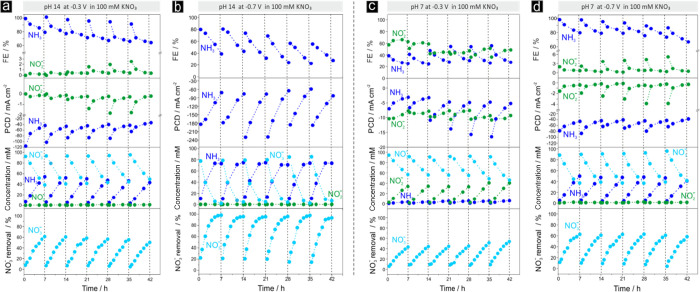
(a) Time-dependent performance data for extended (42 h) discontinuous electrolyses carried out using the Cu-foam(30 s)@mesh sample as the catalyst. (a) pH 14, *E* = −0.3 V vs RHE, *c*_nitrate_ = 100 mmol L^–1^. (b) pH 14, E = −0.7 V vs RHE, *c*_nitrate_ = 100 mmol L^–1^. (c) pH 7, *E* = −0.3 V vs RHE, *c*_nitrate_ = 100 mmol L^–1^. (d) pH 7, *E* = −0.7 V vs RHE, *c*_nitrate_ = 100 mmol L^–1^.

Figure 8 depicts the results of the extended electrolyses at pH 14 (Figure 8a,b) and pH 7 (Figure 8c,d), respectively. Product selectivity data are provided in terms of FEs and PCDs for ammonia and nitrite formation. These data are further complemented by the time-resolved changes in the relevant reactant and product concentrations, as well as the time-dependent nitrate removal efficiency. In the literature, the latter is often considered an important metric for the NO_3_^–^RR performance evaluation,^65^ although this quantity strongly depends on the specific dimensions of the electrolysis cell and the surface area of the catalyst. Consequently, any comparison of catalyst performance data from different studies on the basis of only this specific metric is difficult.

Both the FE and PCD values for ammonia production demonstrated a strong time dependence resulting from the continuous consumption of the nitrate reactant in the batch reactor over time. Within the first 7 h of electrolysis at pH 14 and −0.3 V vs RHE (Figure 8a), the FE_NH3_ values decreased from 99.1% initially to 79%, in line with the decrease in the nitrate concentration from 100 mmol L^–1^ initially to ∼40 mmol L^–1^ (corresponding to ∼60% of nitrate removal). Importantly, the “initial” FE_NH3_ values, always measured 30 min after the (re)start of the respective 7 h continuous electrolysis, largely recovered after the electrolyte replenishment steps. The “initial” FE_NH3_ value remained stable after the first and second electrolyte replenishment (101.2% at 7.5 h; 97.5% at 14.5 h), then gradually decreased to 95.6% (21.5 h), 94.4% (28.5 h), and finally to 91.2% (35.5 h). Importantly, the corresponding PCDs of ammonia production (PCD_NH3_) followed the observed trend of moderately decreasing FE_NH3_ values. In the initial 30 min catalyst screening experiment at pH 14 (Figure 7), NO_2_^–^ was not observed as a relevant byproduct of NO_3_^–^RR at −0.3 V vs RHE. However, the results presented in Figure 8a demonstrated that the catalyst stress through the repetitive electrolyte replenishment led to minor but clearly visible alterations in product selectivity toward the formation of the undesired nitrite byproduct at the expense of the targeted ammonia formation. After the 5th replenishment (35.5 h), the “initial” FE_nitrite_ value was clearly above 2%. A different scenario was observed for the corresponding extended electrolysis at −0.7 V vs RHE (Figure 8b). Our analysis confirmed that NH_3_ was the prevalent NO_3_^–^RR product at higher overpotentials, whereas NO_2_^–^ formation remained fully suppressed even with the “repetitive” catalyst stress approach. However, the main byproduct of nitrate electrolysis at higher overpotentials (regime (3)) was hydrogen. Under optimum experimental conditions (pH 14, 100 mmol L^–1^ nitrate concentration; see also Figure S21), the NO_3_^–^ removal reached 100% after 7 h of electrolysis even after the 5th electrolyte replenishment step (Figure 8b). A noteworthy difference to the extended electrolysis at −0.3 V vs RHE (Figure 8a) concerns the “initial” FE_NH3_ values determined 30 min after the (re)start of the respective 7 h lasting continuous electrolysis (Table S12), which clearly did not follow the same trend of the “initial” PCD_NH3_ values. The initial value of 79.9% (0.5 h) remained fairly stable after the first electrolyte replenishment (80.5% at 7.5 h), decreased over the course of the subsequent electrolyte exchange steps, and then reached a fairly stable level of 56.5 and 55.5% at 28.5 h (4th replenishment) and 35.5 h (5th replenishment), respectively. The corresponding PCDs for ammonia production appeared to be decoupled from this trend. At an electrolysis time of 0.5 h, the first determined PCD_NH3_ value was −183.9 mA cm^–2^. After the 5th electrolyte replenishment, the “initial” PCD_NH3_ values reached a nearly identical value of −187.4 mA cm^–2^, thereby indicating that the ammonia production rate was unexpectedly not affected by the overall downward trend of the “initial” FEs for ammonia. We assume that the origin of this remarkable effect of decoupled FE_NH3_ and PCD_NH3_ values was due to the experimental conditions applied, wherein the NO_3_^–^RR was already mass transport limited and therefore was affected, i.e., accelerated, by the gas-evolving HER superimposed on the NO_3_^–^RR. A decrease in the initial FE_NH3_ values suggested a corresponding anti-correlated increase in the initial FE_H2_ values.

**Figure 9:**
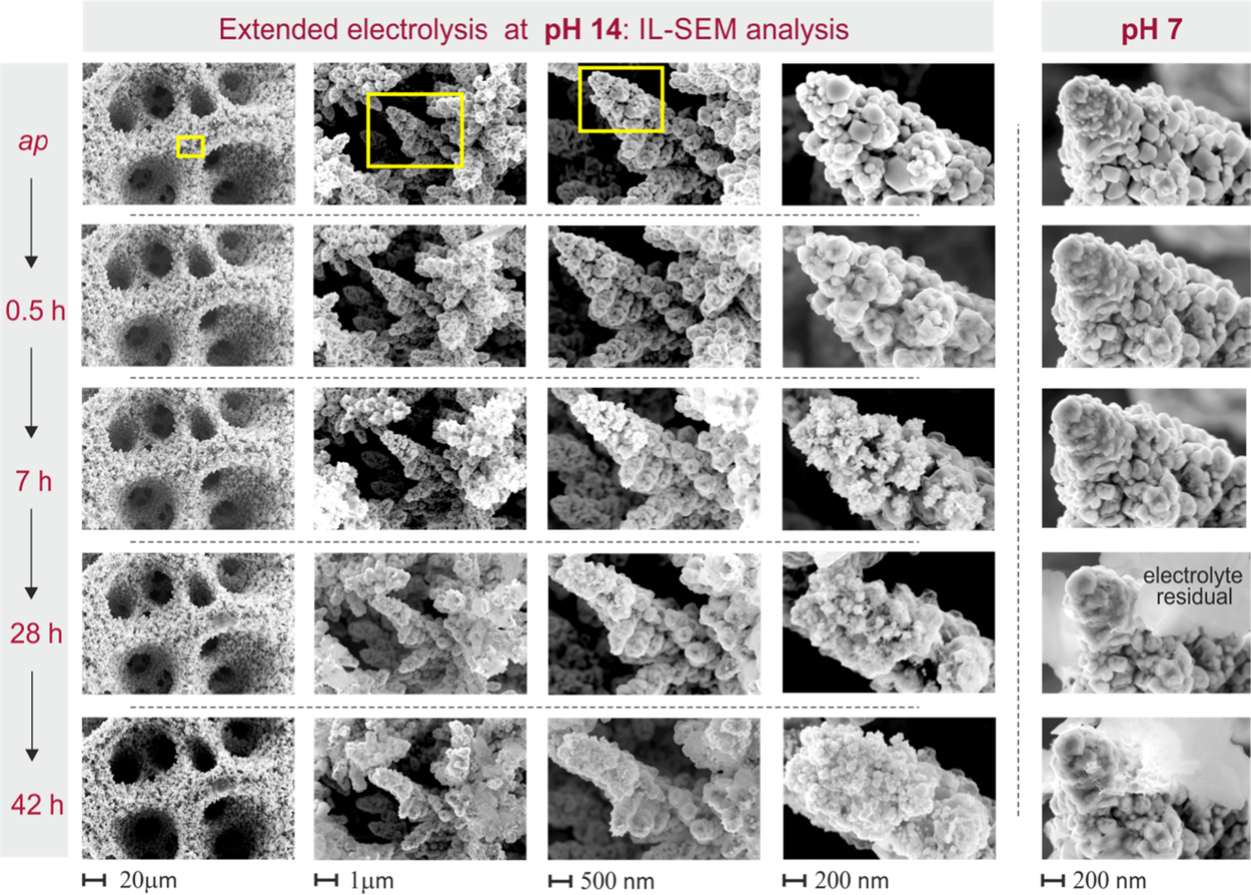
Representative identical location (IL) SEM analysis of the Cu-foam(30 s)@mesh catalyst as a function of the electrolysis time. The electrolysis was carried out at *E* = −0.7 V vs RHE in the 100 mM KNO_3_ (pH 14) electrolyte. For the IL-SEM analyses, the electrolysis was interrupted at dedicated electrolysis times as indicated in the figure. For comparison purposes, also the high-resolution IL-SEM data for the corresponding electrolysis at pH 7 are shown.

Moreover, the nanoscale surface morphology of the Cu foam catalyst experienced alterations in the extended stress experiment at −0.7 V vs RHE according to the appearance of smaller nanometer-sized Cu particles on top of individual Cu dendrites. These structural changes were visualized in complementary identical location SEM analyses performed after completion of the 7 h continuous electrolyses. This increase in the nanoscale surface roughness and the related creation of additional under-coordinated surface sites might have been the origin of the observed trend toward favored HER (Figure 9). However, the macroporosity of the Cu-foam(30 s)@mesh remained fully unaffected by the extended electrolysis at pH 14 and −0.7 V vs RHE, although the total current density reached values beyond −336 mA cm^–2^. This finding demonstrated that the foam morphology was generally suited for electrolyses performed at high current densities and involving hydrogen gas bubble formation. On the basis of these considerations, NO_3_^–^RR performance losses, in terms of the observed downward trend in the FE_NH3_ values, can be concluded to be counterbalanced by improved nitrate mass transport through more intense hydrogen gas bubble formation. Consequently, the initial PCD_NH3_ values remain high despite the losses in ammonia FE.

The excellent long-term durability of the Cu-foam(30 s)@mesh catalyst was also evidenced by the corresponding extended electrolyses performed at pH 7 (Figure 8c,d). The NO_3_^–^ removal determined after completion of the individual 7 h long continuous electrolysis steps was only rarely affected by the repetitive electrolyte replenishments and related catalyst surface oxidation/reduction processes (Table S13). These results confirmed that NO_2_^–^ remained the prevalent NO_3_^–^RR product of electrolysis at −0.3 V vs RHE and pH 7 (Figure 8c) even in the extended discontinuous stress experiment. Nitrite formed at a fairly constant (low) rate (PCD_nitrite_), which was only marginally affected by the repetitive catalyst stress. Extended catalyst stress performed at −0.7 V vs RHE (Figure 8d) confirmed that the potential window in which nitrite forms as a byproduct was substantially broadened at pH 7. Again, repetitive catalyst stress led only to rather minor changes in the product distribution slightly favoring nitrite over time.

**Figure 10:**
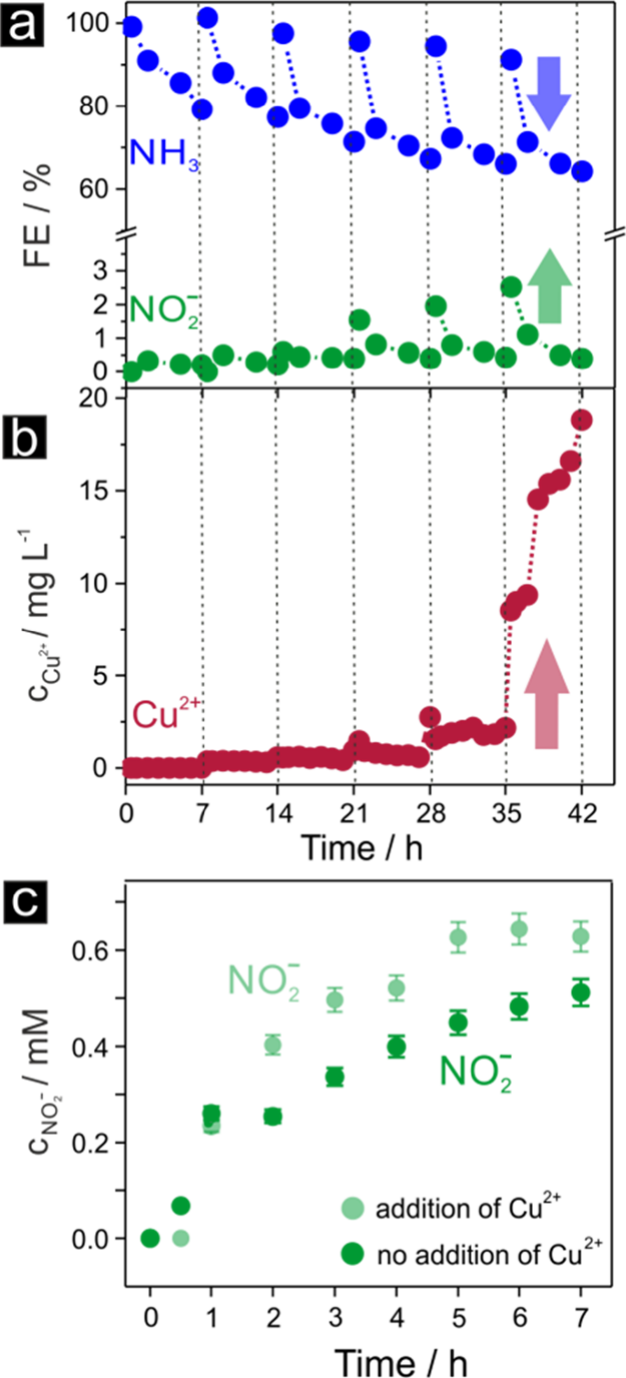
Intermediate formation of cupric (Cu^2+^) ions as origin for the observed losses in the nitrate → ammonia efficiency. (a) Representative time-dependent performance data for extended (42 h) discontinuous electrolyses carried out using the Cu-foam(30 s)@mesh sample as the catalyst at pH 14, *E* = −0.3 V vs RHE, and *c*_nitrate_ = 100 mM L^–1^. (b) Corresponding time-dependent ICP-MS elemental analysis of the catholyte. (c) Control electrolysis experiment (7 h continuous electrolysis) carried out in the (initial) absence and presence of cupric ions (15 ppm) proving an increased formation of nitrite when Cu^2+^ is present.

Reasons for catalyst degradation can be manifold and may include changes in the nano-morphology (structure of active sites and their surface density) and chemical poisoning of the active sites. One important contributor driving product selectivity toward nitrite is the presence of cupric ions in solution. Previous studies have reported that Cu(II) accelerates the rate NO_3_^–^RR;^61,66^ however, that effect was not confirmed in the present study. Of note, Cu(II) ions in solution inevitably undergo electroreduction under NO_3_^–^RR conditions and consequently might affect the ECSA. ECSA changes might have been misinterpreted in the past as Cu(II)-mediated catalytic effects.^66^Figure 10 correlates the trend of increasing nitrite efficiency observed at pH 14 and −0.3 V vs RHE with the concentration cupric ions in the electrolyte solution. As evidenced by ICP-MS analysis, metal ions were released into the electrolyte solution during the reduction of the formed surface oxides during the NO_3_^–^RR—a phenomenon extensively observed in online ICP-MS corrosion studies.^67^ From systematic online ICP-MS studies on the oxidation/reduction of various metals, metal ions are well known to be preferentially released rather during metal-oxide reduction than during oxidative formation.^67^ Additional control experiments in which Cu(II) ions were intentionally added to the electrolyte solution confirmed a larger trend toward nitrite formation in the presence of the cupric ions in the electrolyte solution (Figure 10c).

## Conclusions

High surface area Cu foams electrodeposited on a mesh support electrode (denoted Cu-foam@mesh) with the DHBT approach demonstrated excellent performance in electrochemical nitrate → ammonia conversion, in terms of not only the FEs and N-selectivity, which reached near unity values within a broad parameter space, but also the obtained PCDs for ammonia production, e.g., PCD_NH3_ ≈−1 A cm^–2^ in a 500 mmol L^–1^ KNO_3_ solution at pH 14. Concentration and pH-dependent analyses suggested the preferential formation of parasitic nitrite only at low overpotentials and near neutral pH, whereas undesired nitrite formation remained largely suppressed under strongly alkaline conditions and higher overpotentials. A comparison with ideally planar Cu wafer coupon surfaces confirmed for the porous Cu foam catalyst at pH 14 effective “trapping effects”, thus mitigating the undesired release of nitrite, at least at medium (100 mM) and low (10 mM) nitrate concentrations.

Long-term electrolyses in combination with catalyst stress through (intentional) potential losses and related surface oxidation/reduction processes demonstrated high stability of the Cu-foam(30 s)@mesh against chemical and structural degradation, thus making it a potential catalyst candidate for future nitrate → ammonia conversion processes. The presence of Cu(II) species in solution was demonstrated to be detrimental by shifting the product distribution away from ammonia toward nitrite.

The most important outcome of the present study relates to the effects of the HER which can be superimposed on the NO_3_^–^RR at higher applied overpotentials. When the NO_3_^–^RR becomes mass transport limited, e.g., owing to low reactant concentrations in combination with high conversion rates, the hydrogen gas evolution opens an additional convectional nitrate mass transport pathway, thereby mitigating reactant depletion inside the porous catalyst and boosting the nitrate → ammonia limiting current. These results clearly demonstrate that the HER is not solely a parasitic side reaction of cathodic transformations but can become an inherent part of the catalyst concept, particularly when highly porous three-dimensional catalyst materials are used. Effective reactant transport into the pores of the catalyst foam is a conceptional prerequisite for realizing the benefits of this high surface area catalyst material. Forthcoming studies using dedicated electrolyte flow cells and inverted RDE configurations for easy gas release will further reveal the effect of forced convection on the reactant replenishment inside of such highly porous foam catalysts under stationary mass transport conditions. These future studies will also allow for a comparison and discussion of NO_3_^–^RR process energy efficiencies by considering also the energy input required for other means of forced electrolyte convection (laminar electrolyte flow, RDE configuration, etc.).

## Data Availability

The raw data to this paper are made fully accessible to the public via Zenodo doi:10.5281/zenodo.7446294 along with the publication of this manuscript.

## Supporting Information Available

Optical micrographs, SEM images, ion and cyclic voltammograms, reference measurements, and calibration data (PDF)
Cu wafer_ NO_3_^–^RR (MP4)
NO_3_^–^RR _Cu foam (MP4)
