# Supplementary material for: Boosting Nitrate to Ammonia Electroconversion through Hydrogen Gas Evolution over Cu-foam@mesh Catalysts
Source: ACS Catal. 2023 Jun 5;13(12):8169–82. doi: 10.1021/acscatal.3c00716 (PMC10278070; doi:10.1021/acscatal.3c00716)
Supplement: Supplementary file 1 — cs3c00716_si_001.pdf [file cs3c00716_si_001.pdf]

Supporting Information

Boosting Nitrate to Ammonia Electroconversion through Hydrogen Gas Evolution over Cu-foam@mesh Catalysts

Yuzhen Wang[1,2], Abhijit Dutta[1,3]*, Anna Iarchuk[1, 3], Changzhe Sun[1], Soma Vesztergom[1,3, 4], and Peter Broekmann[1,3]*

[1]Department of Chemistry, Biochemistry and Pharmaceutical Science, University of Bern, Freiestrasse 3, 3012 Bern, Switzerland

[2]State Key Laboratory of Eco-hydraulics in Northwest Arid Region of ChinaXi'an University of Technology, No.5 South Jinhua Road, Xi'an, Shaanxi,710048, China

[3]National Centre of Competence in Research (NCCR) Catalysis, University of Bern, Freiestrasse 3, 3012 Bern, Switzerland

[4]Eötvös Loránd University, MTA–ELTE Momentum Interfacial Electrochemistry Research Group, Pázmány Péter sétány 1/A, 1117 Budapest, Hungary

**Corresponding authors: abhijit.dutta@unibe.ch (A. Dutta) and peter.broekmann@unibe.ch (P. Broekmann)*

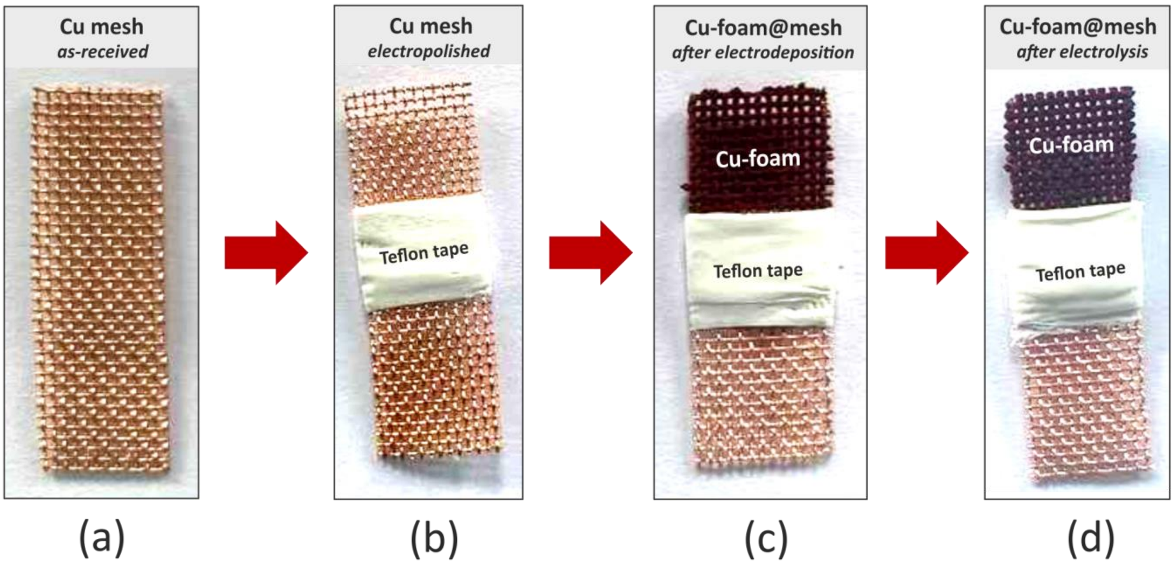

Figure S1. a) – d) Photographs showing consecutive process steps applied for the preparation and application of the Cu-foam@mesh catalyst.

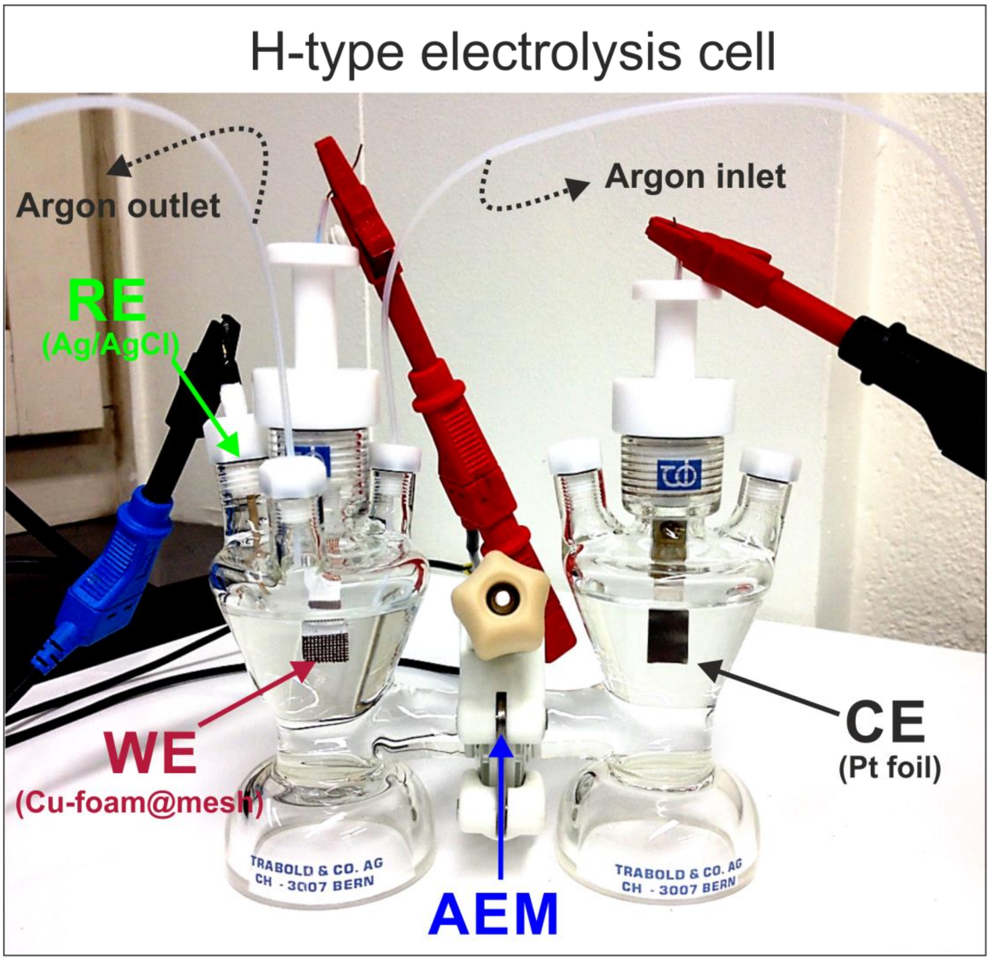

Figure S2. Optical micrograph of the (divided) H-type electrolysis cell used in this study.

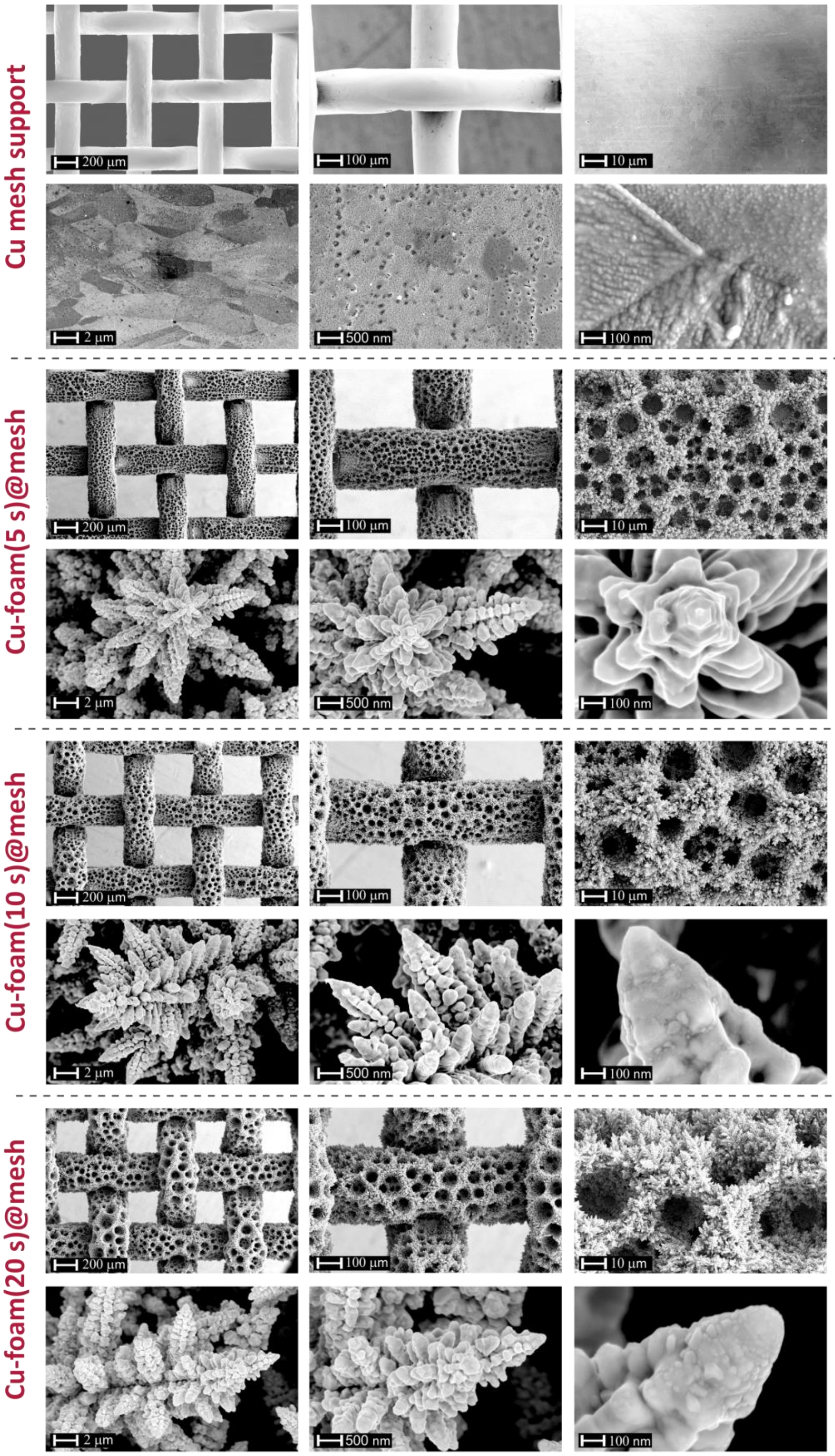

Figure S3 (part 1). SEM images of increasing magnification showing the evolution of the multilevel porosity of the Cu-foam@mesh catalysts depending on the deposition time ranging from 5 to 20s. For comparison purposes also the morphology of the electropolished Cu mesh is provided in the upper panel.

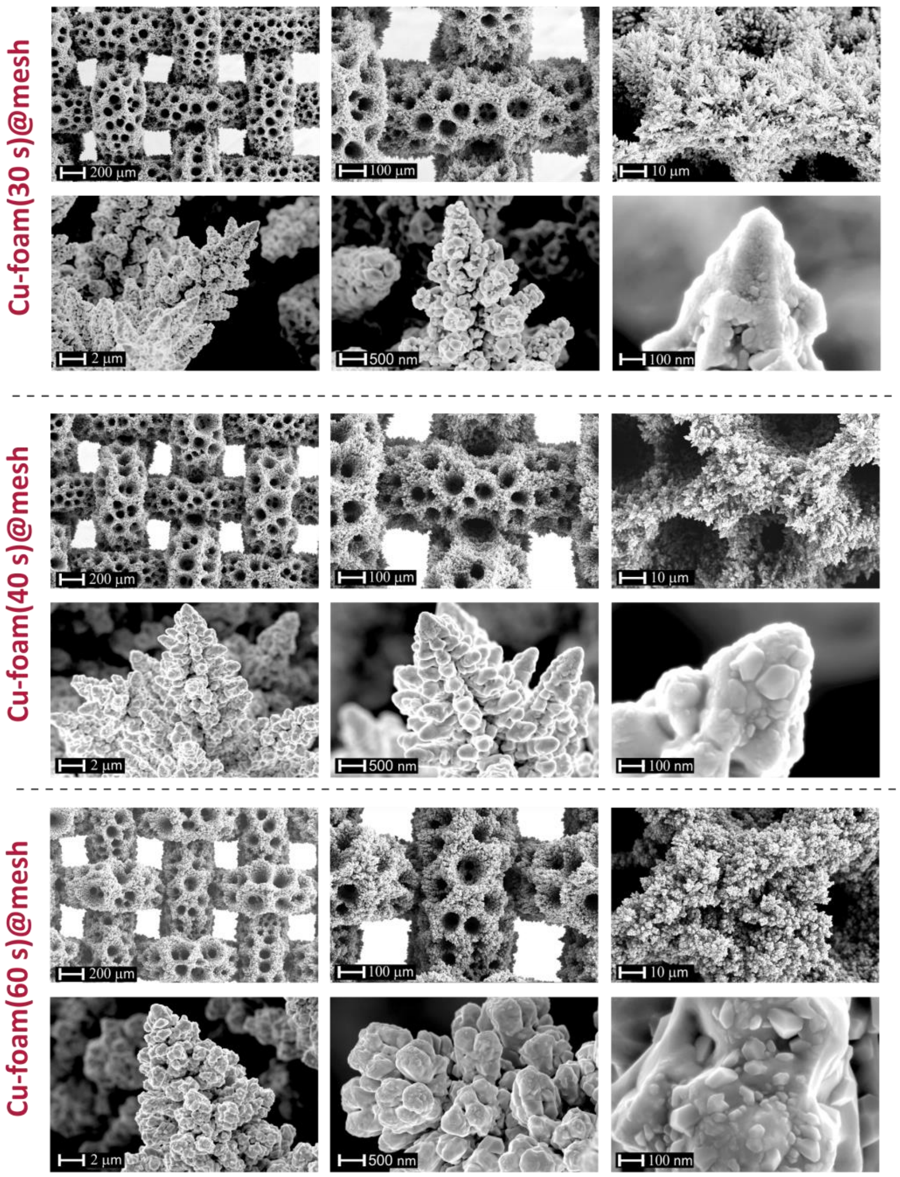

Figure S3 (part 2). SEM images of increasing magnification showing the evolution of the multilevel porosity of the Cu-foam@mesh catalysts depending on the deposition time ranging from 30 to 60 s.

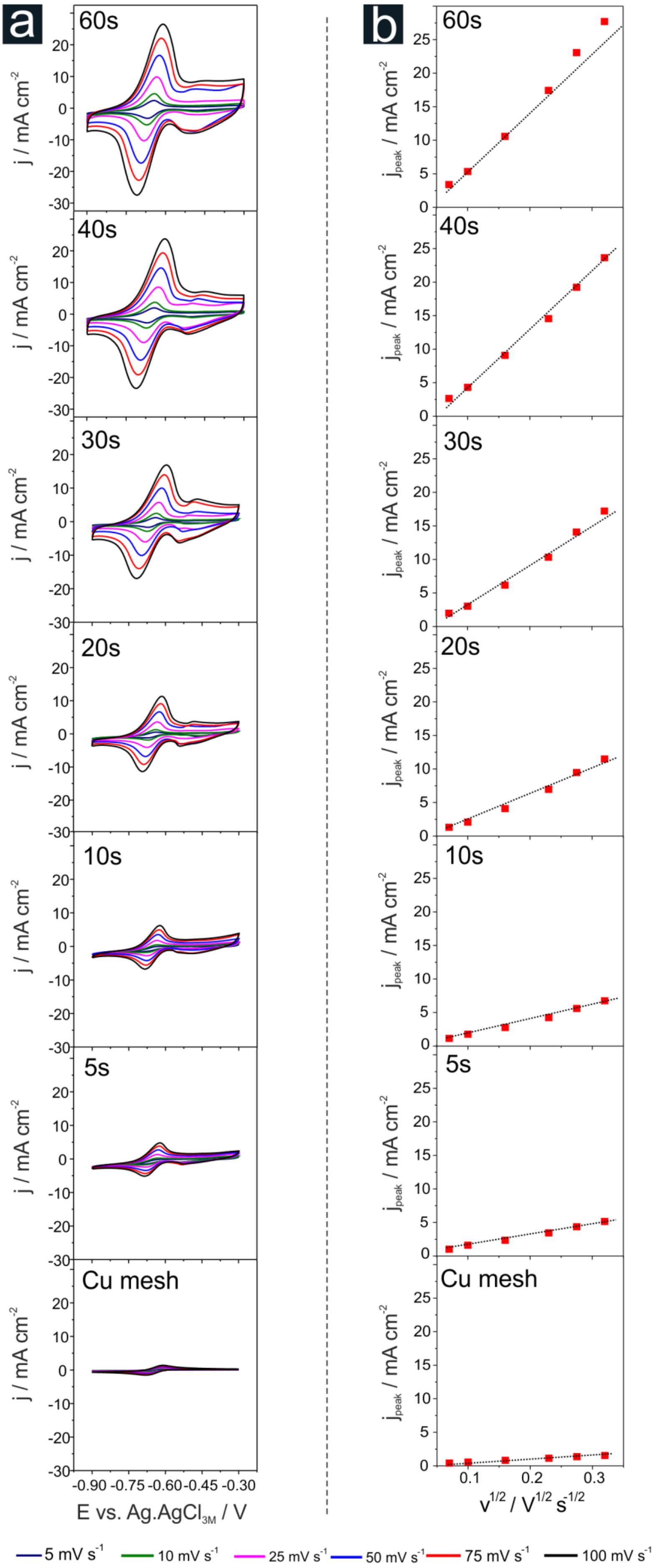

Figure S4. a) Sweep rate dependent cyclic voltammograms (CVs) of the Cu mesh (reference/support) and the electrodeposited Cu foams (5 – 60 s deposition time) measured in an electrolyte solution containing 10 mM dimethyl viologen dichloride (DMVCl$_2$; Sigma Aldrich, 98%) as reversible redox probe and 1 mol L^{-1} Na$_2$SO$_4$ (Sigma Aldrich, ≥99.0) as the supporting electrolyte. Note, the current densities were normalized to the geometric surface area. b) Plots of the (reduction) peak current densities (j_{peak}) versus the square root of the potential sweep rate ($\nu^{1/2}$). The ECSA values (Table S1) were estimated and derived from the slope of the corresponding linear regressions (application of the Randles-Ševčík equation).

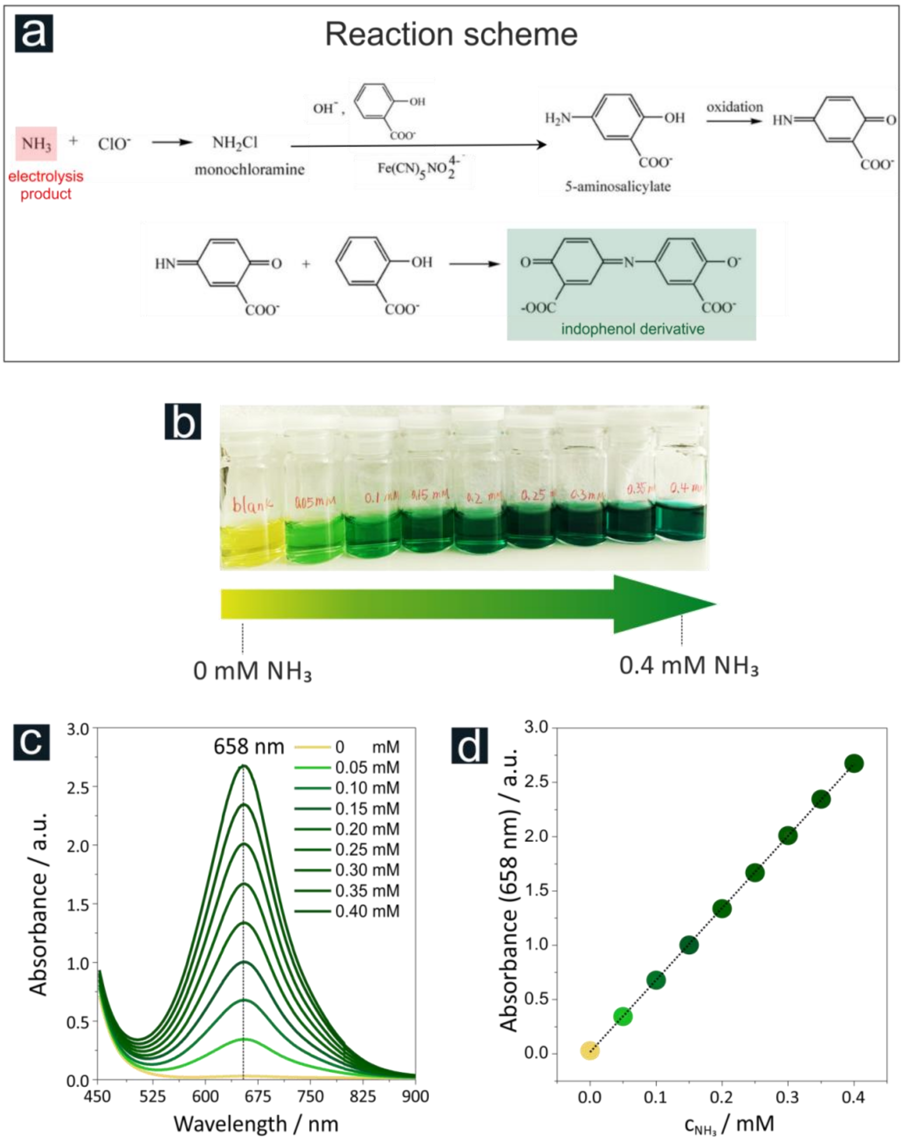

Figure S5. a) Reaction scheme showing the transformation of ammonia (targeted NO_3^-RR product) into the indophenol derivative which was then detected and quantified by means of UV-visible absorption spectroscopy. b) Optical micrographs showing a dilution series of solutions used to produce the calibration curve. c) Corresponding UV-visible absorption spectra. d) Calibration curve produced on the basis of the UV-visible absorption spectra shown in Figure 5c.

Ammonia quantification: aliquots of the catholyte were diluted 20 to 100 times with Milli-Q water. Afterward, 2 mL of the diluted catholyte was mixed with 1 mL 0.05 mol L^{-1} $NaClO_4$ (Sigma-Aldrich, reagent grade), 2 mL of 1 mol L^{-1} NaOH solution (Sigma-Aldrich, >98.0%) containing 5 wt.% salicylic acid (Sigma Aldrich, ≥99.0%) and 5 wt.% sodium citrate (Sigma Aldrich, ≥99.0%), and 200 μL of 1 wt.% sodium nitroferricyanide solution (Sigma Aldrich, ≥99%). After a reaction time of ~1 hour, UV-visible absorption spectra were recorded in the range from 450 to 900 nm with a PerkinElmer Lambda 900 UV-visible/NIR spectrometer. The characteristic absorption maximum of the indophenol derivative was observed at a wavelength of λ = 658 nm (see Figure S5c). The ammonia quantification through the formed indophenol derivative was based on calibration curves (see Figure S5d).

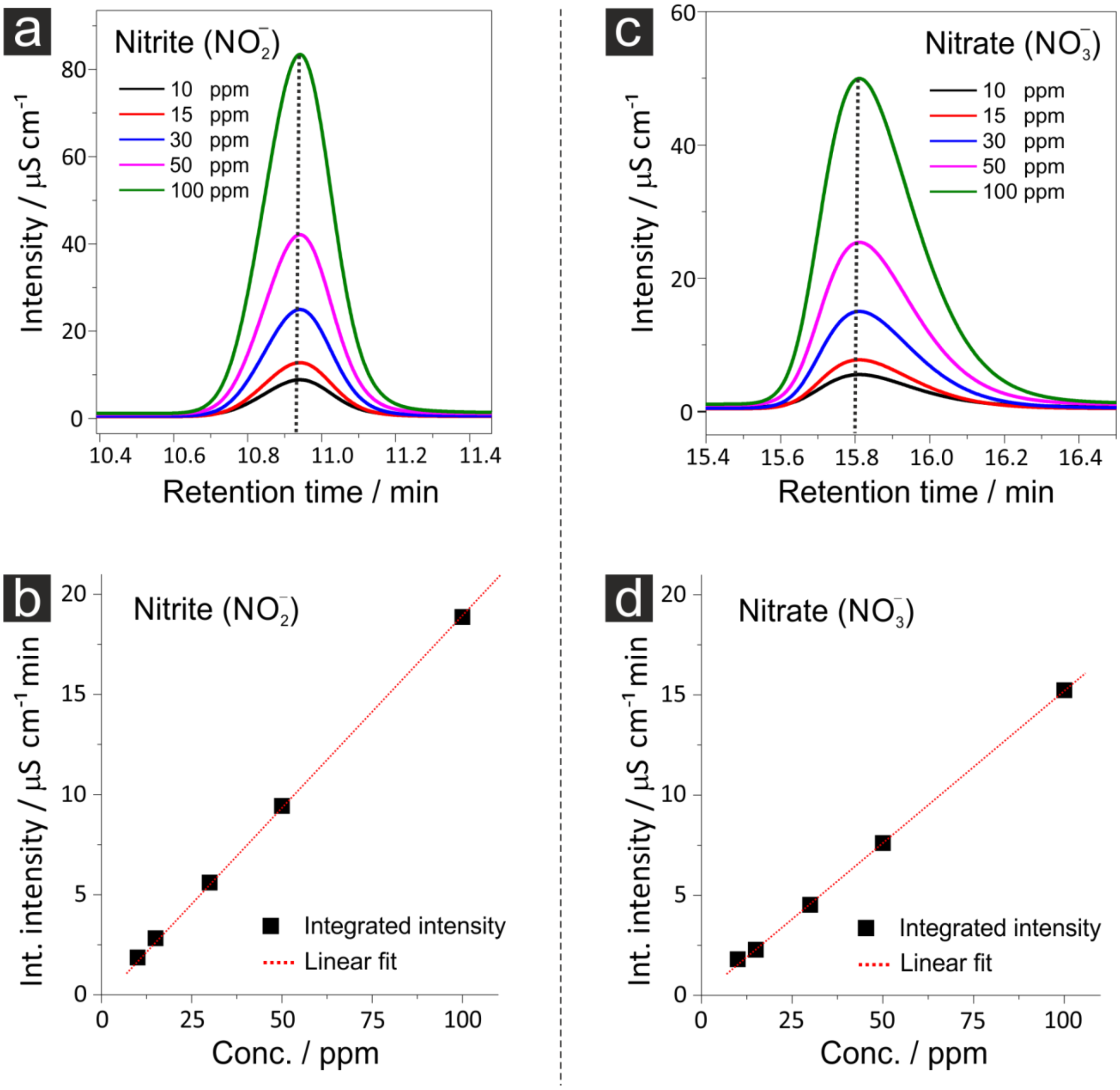

Figure S6. a) Ion-chromatograms of nitrite reference samples in the concentration range from 10 – 100 ppm. b) Resulting linear calibration curve used for the nitrite quantification. c) Ion-chromatograms of nitrate reference samples in the concentration range from 10 – 100 ppm. d) Resulting linear calibration curve used for the nitrate quantification.

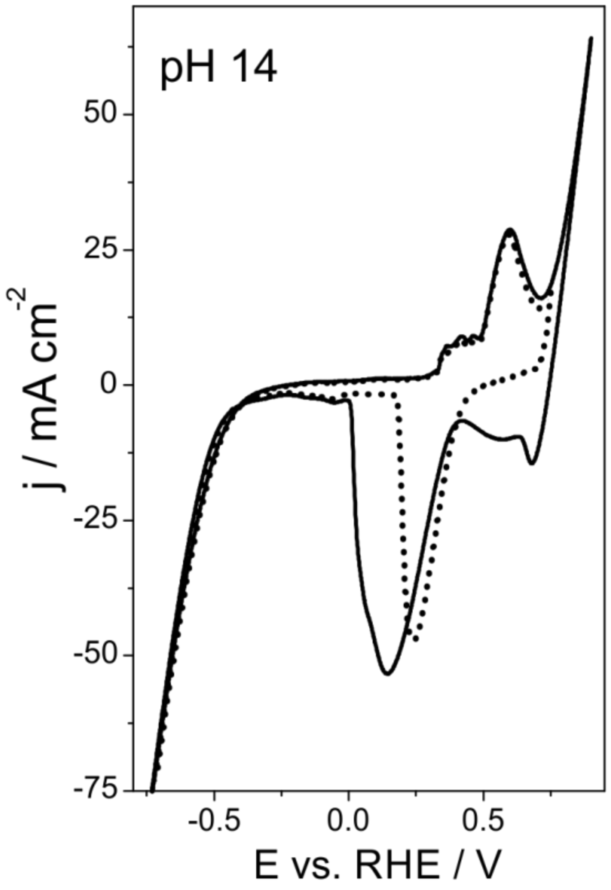

Figure S7. Cyclic voltammograms recorded in the blank supporting electrolyte solution at pH 14 (1 mol L^{-1} KOH) at a sweep rate of 25 mV s^{-1}. Shifting the anodic vertex potential further into the potential range of passive film break down and anodic Cu dissolution leads to a substantial broadening of the main cathodic reduction feature in the reverse potential sweep associated to the passive film reduction.

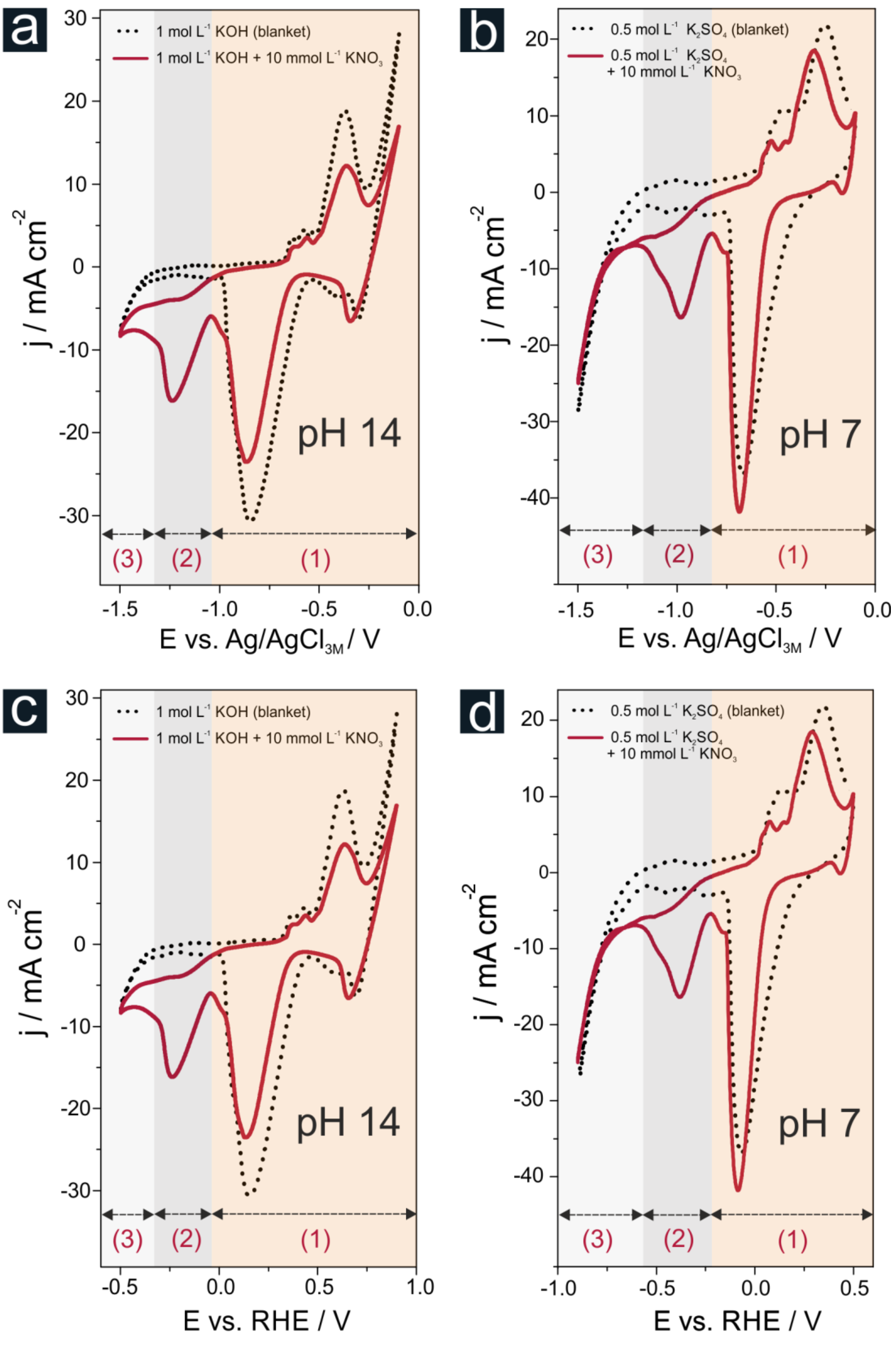

Figure S8. Cyclic voltammograms recorded in nitrate containing and nitrate-free electrolytes at pH 14 and pH 7. a) Measured at pH 14 versus $Ag/AgCl_{3M}$. b) Measured at pH 7 versus $Ag/AgCl_{3M}$. c) Measured at pH 14 and referenced versus RHE. d) Measured at pH 7 and referenced versus RHE. Three characteristic regimes are highlighted: (1) Cu_xO formation/reduction, Cu dissolution/re-deposition; (2) NO_3^-RR; (3) NO_3^-RR superimposed to the HER. The potential sweep rate was in all cases 25 mV s^{-1}. The working electrode was the Cu-foam(30 s)@mesh sample.

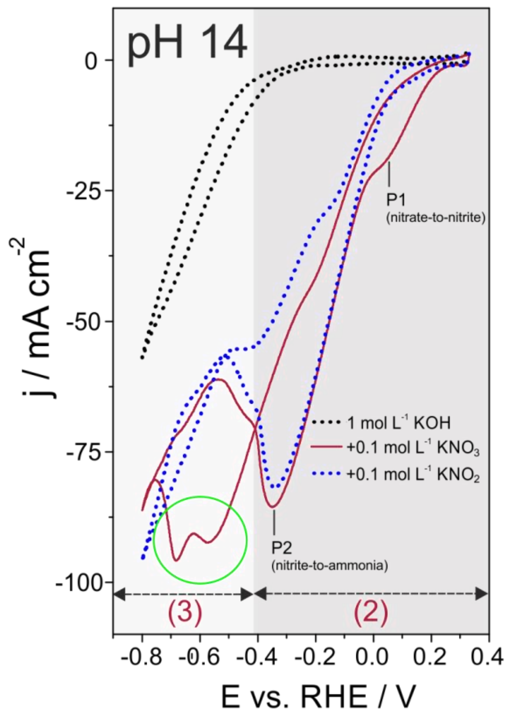

Figure S9. Cyclic voltammograms recorded at pH 14 in the blank supporting electrolyte solution (1 mol L^{-1} KOH) and in solutions containing either 100 mmol L^{-1} KNO_3 or 100 mmol L^{-1} KNO_2. The potential sweep rate was in both cases 25 mV s^{-1}. The working electrode was the Cu-foam(30 s)@mesh sample. For these control experiments an anodic vertex potential was chosen more negative than the onset of Cu oxidation. The comparison of the CVs recorded in the nitrate and nitrite containing electrolytes confirms that the first cathodic current feature **P1** originates from the nitrate$\rightarrow$nitrite reduction whereas the main current peak **P2** is due to the further reduction of the formed nitrite into ammonia. Note that P1 is absent in the voltammogram when nitrite is the reactant. Highlighted in green are extra cathodic current features often appearing in the reverse potential sweep. These (artifacts) are, however, due to fluctuation of the cathodic currents due to hydrogen bubble formation and release (not reproducible!). These fluctuations occur because of massive gas evolution thus leading to sudden changes of the active surface area and the hydrodynamic conditions.

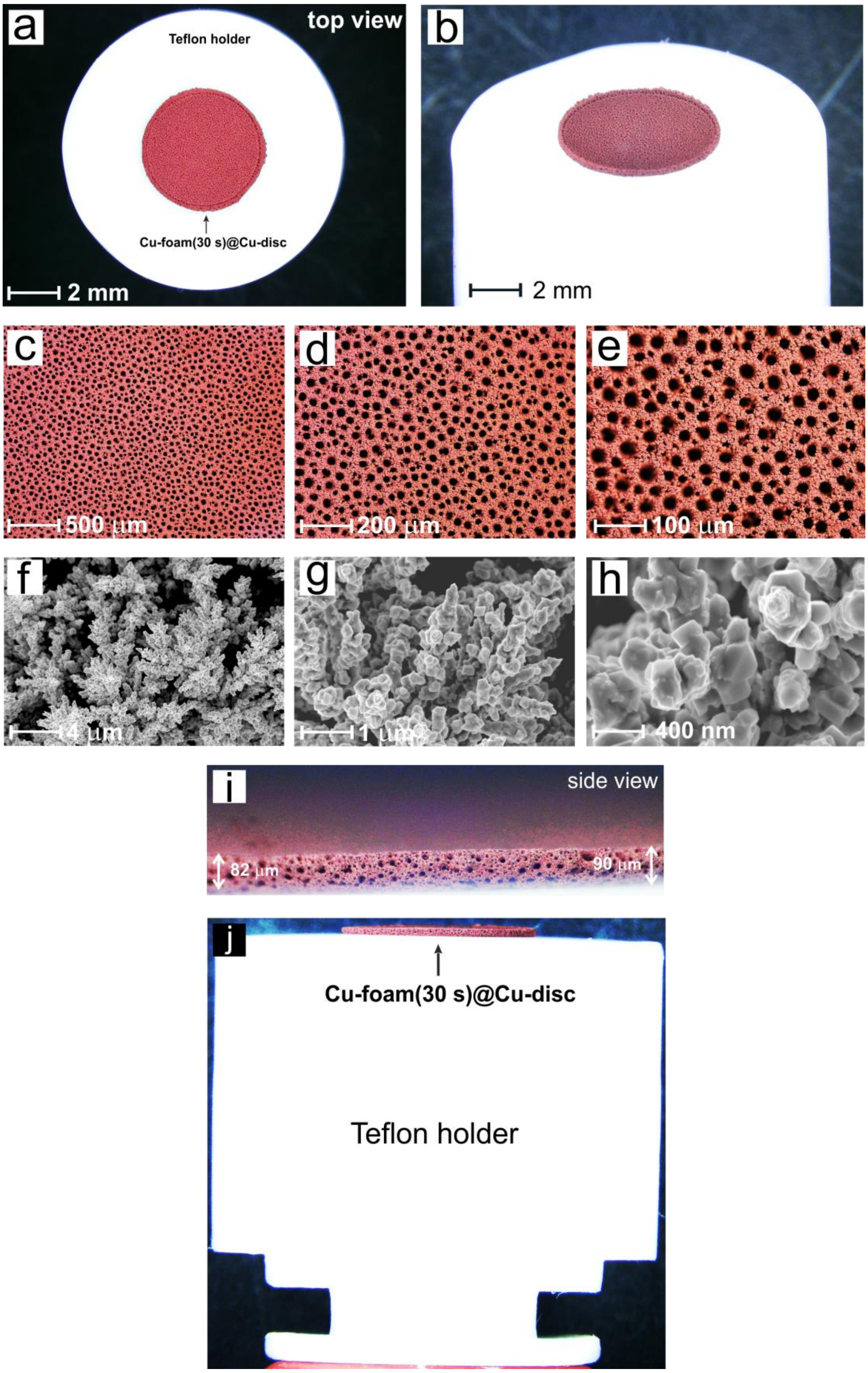

Figure S10. a-e) Optical micrographs of the Cu-foam(30 s)@disk catalyst prepared for RDE reference measurements. f-h) Scanning electron microscopic (SEM) inspection of the Cu-foam(30 s)@disk catalyst. i-j) Optical micrographs (side-view) of the Cu-foam (30 s) on the Cu disc embedded into the Teflon RDE holder.

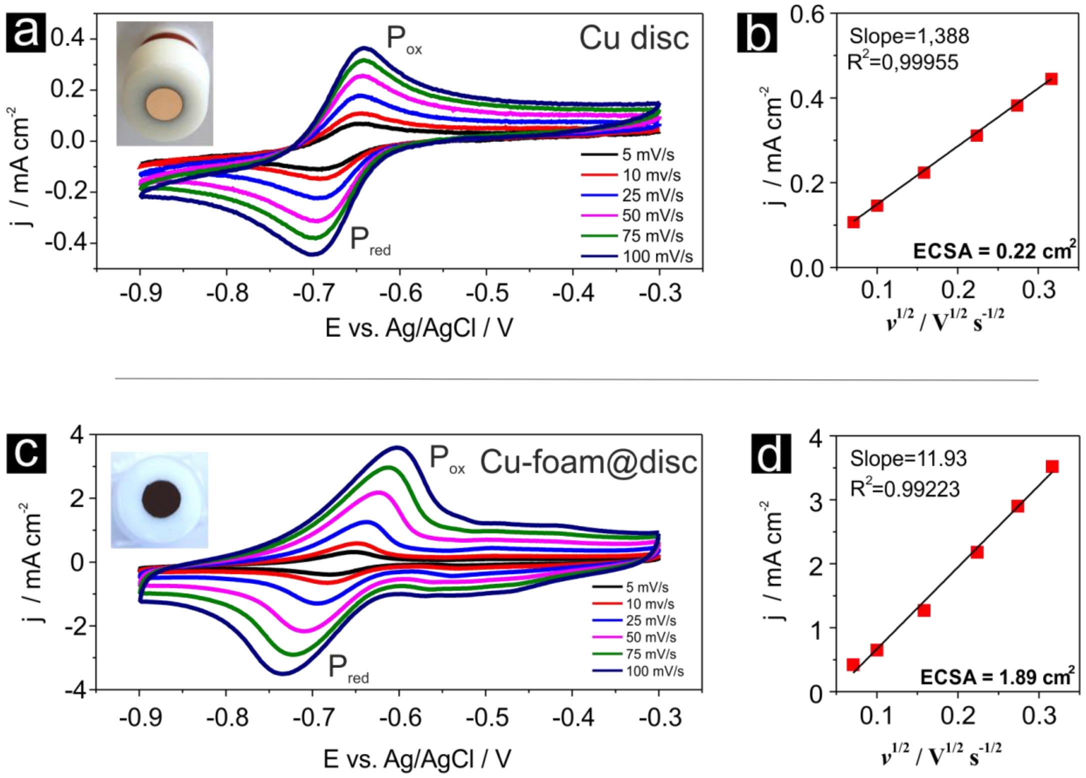

Figure S11. ECSA determination using the viologen method (see Experimental section and Figure S4). a-b) Data obtained for the Cu disc. c-d) Data obtained for the Cu-foam(30 s)@disc sample.

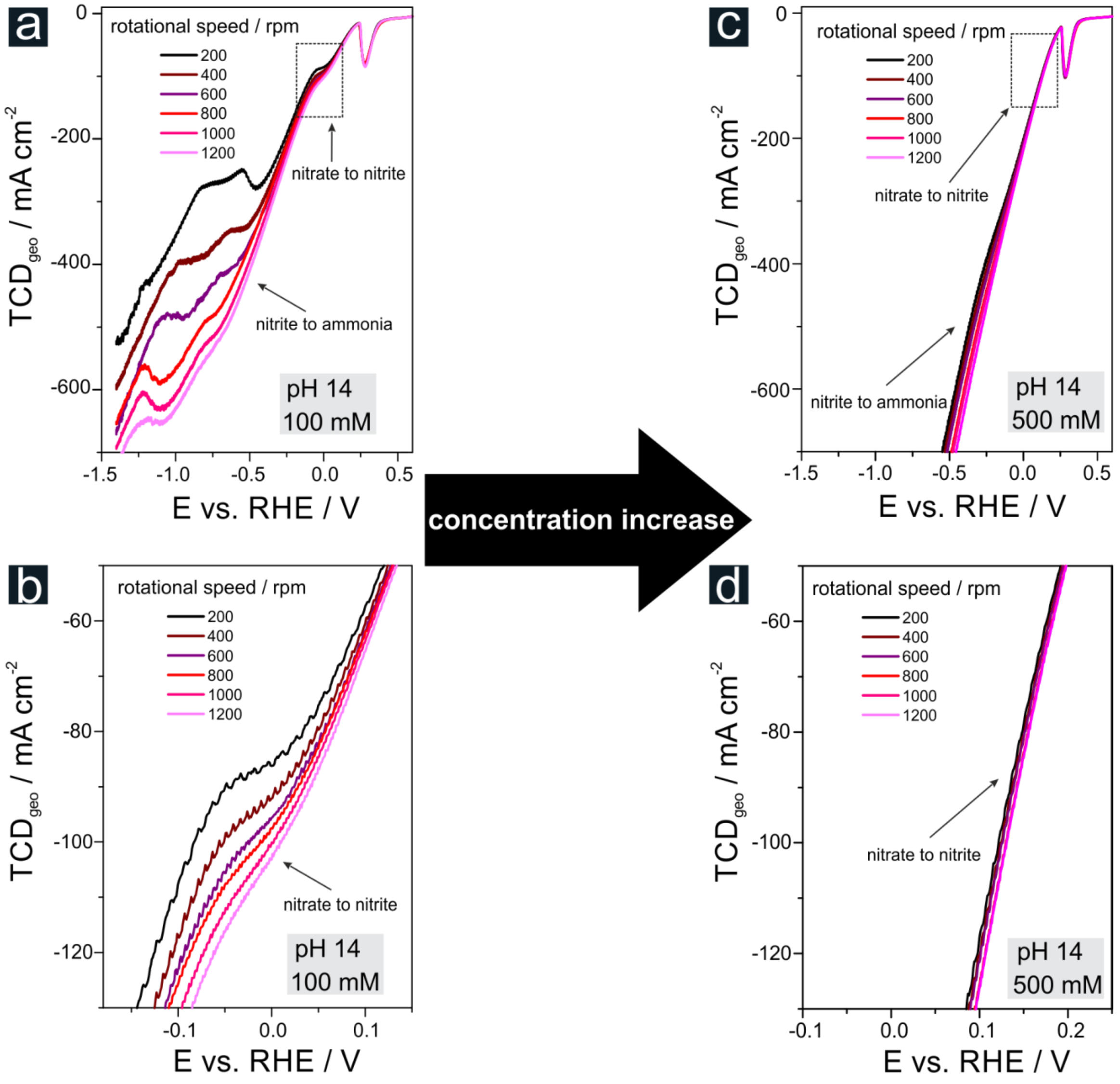

Figure S12. a-b) Angular frequency (rotational speed) depending linear sweep voltammograms (LSVs) demonstrating nitrate mass transport limitations for both the nitrate→nitrite and the nitrate→ammonia transformation at pH 14 (100 mmol L^{-1} nitrate concentration). c-d) Corresponding RDE data for pH 14 using a nitrate concentration of 500 mmol L^{-1}. Obviously mass transport limitations are omitted at these higher nitrate concentrations.

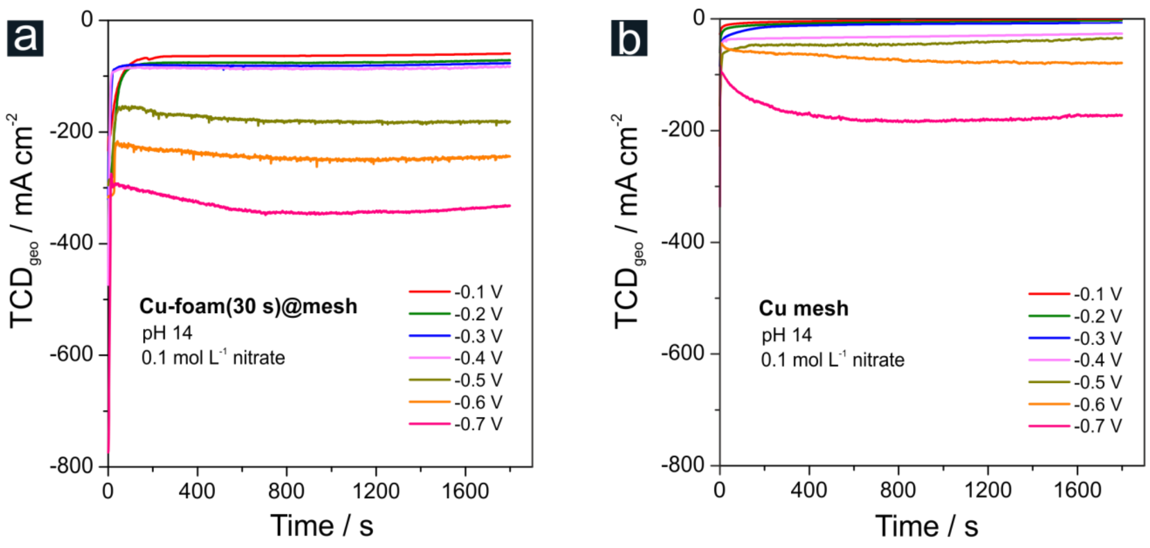

Figure S13. Representative chrono-amperometric data (total current density (TCD_{geo}) versus time plots) corresponding to Figure 5d. Note that the electrolysis performance data presented in Figure 5 is derived from three independent electrolyses per applied potential.

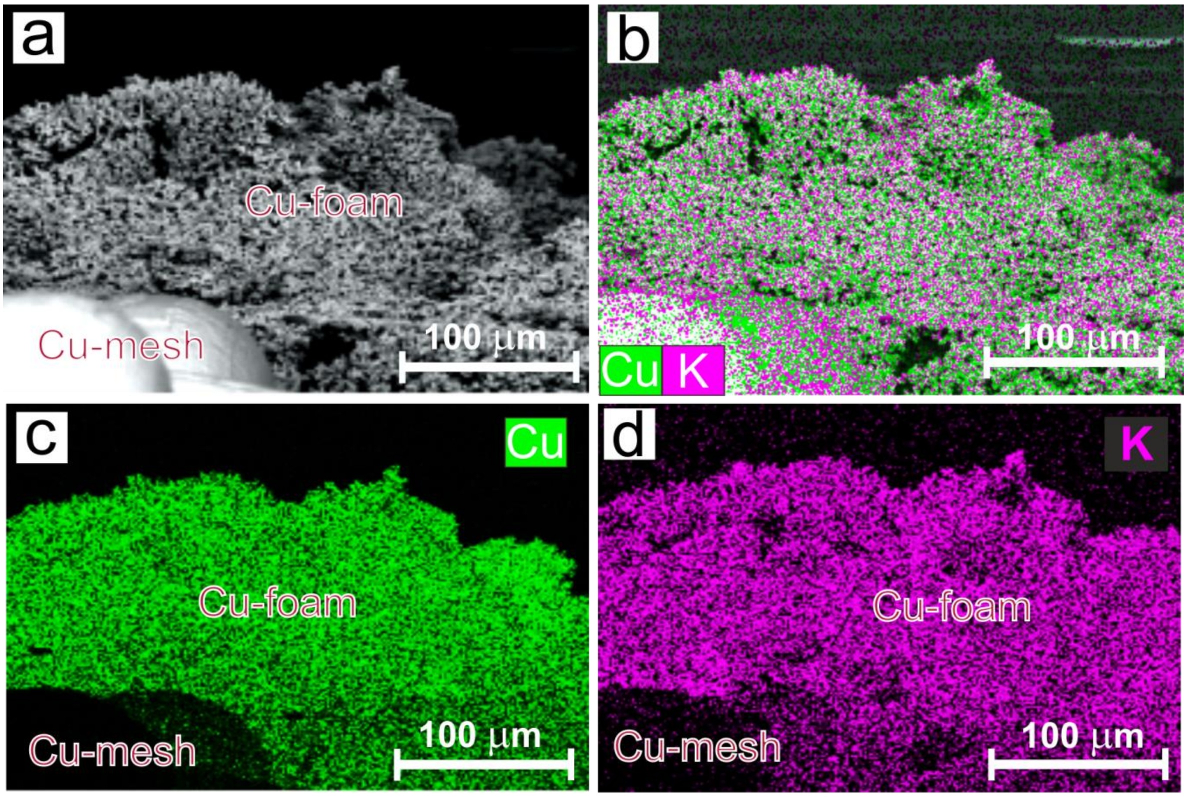

Figure S14. a) Cross-sectional SEM image of the Cu-foam(60 s)@mesh sample after electrolysis at -0.3 V vs. RHE (see Figure 5a-c). b) Superimposed SEM-EDX-K/Cu mapping. c) EDX-Cu mapping. d) EDX-K mapping.

This SEM/EDX inspection confirms the complete wetting of the Cu-foam(60 s)@mesh sample with electrolyte down to the mesh support.

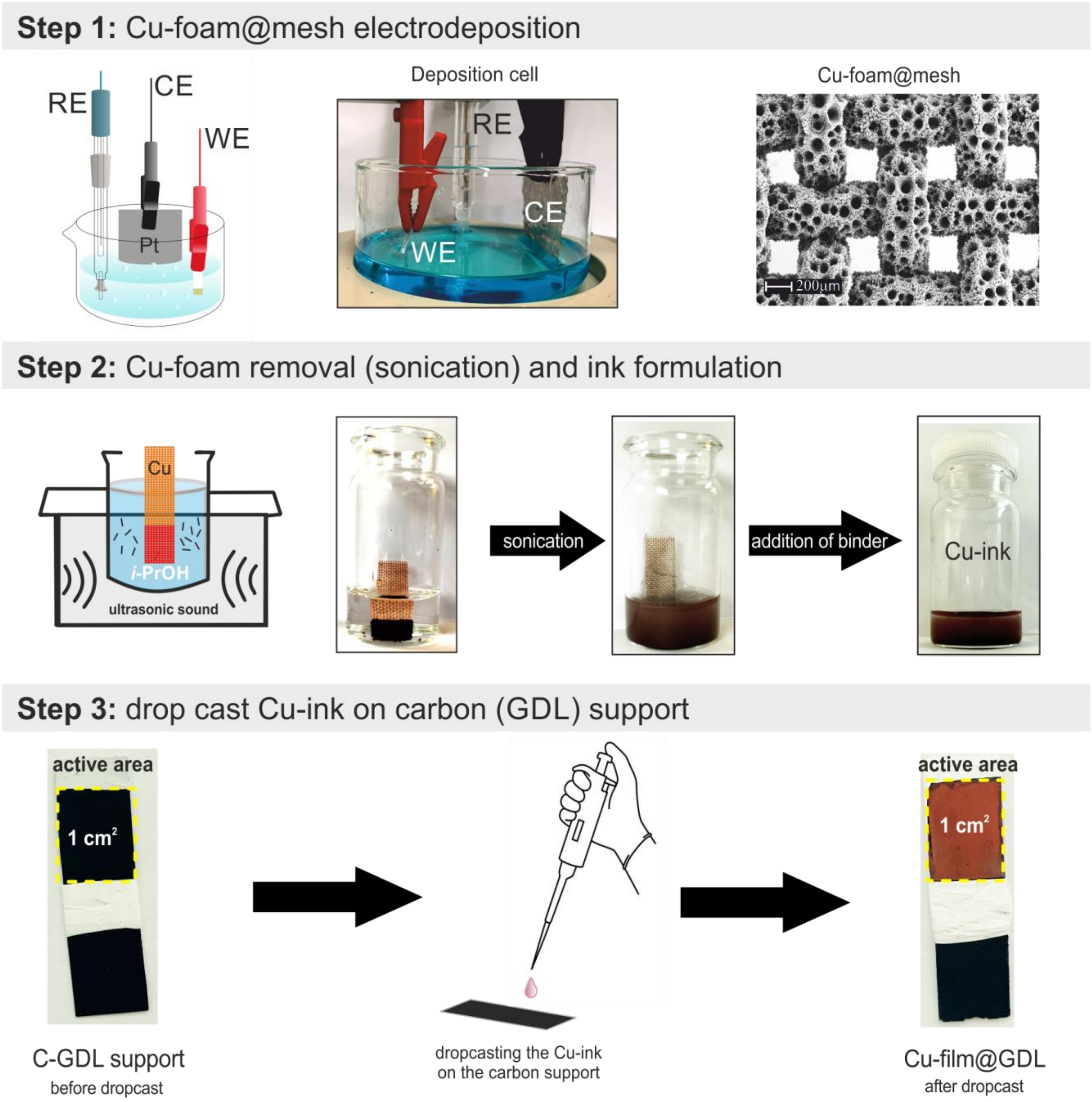

Figure S15. Individual steps applied for the preparation of the Cu film catalyst derived from the Cu-foam(30 s)@mesh sample (see also Experimental section).

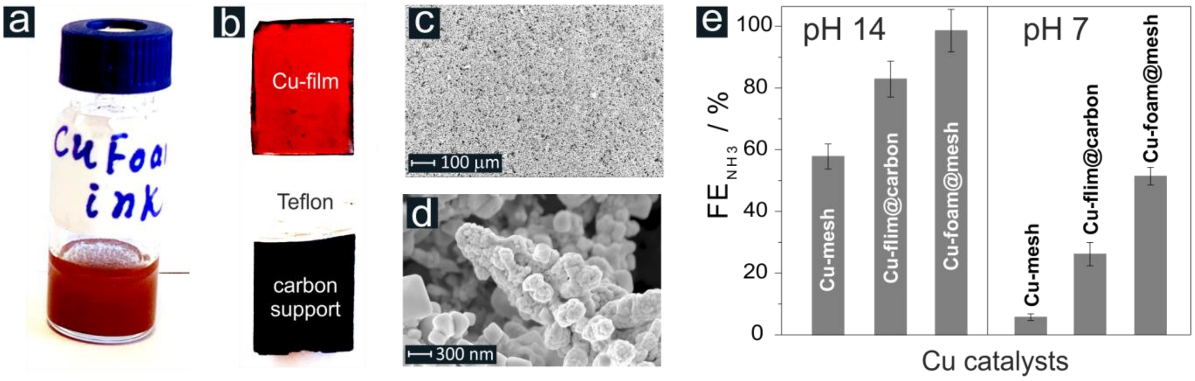

Figure S16. a) Catalyst ink formed from the dendritic Cu removed from the Cu-foam(30 s)@mesh samples by ultrasonication (see Experimental section and Figure S15). b) Optical micrograph of the dendritic Cu film catalysts deposited on a carbon support. c-d) SEM micrographs showing the Cu film catalyst on a µm (c) and nm length scale (d). e) Detected ammonia Faradaic efficiencies after 30 min of electrolysis at –0.3 V vs. RHE. The Cu-foam(30 s)@mesh catalysts outperform at pH 14 (1 mol L^{-1} KOH + 100 mmol L^{-1} KNO_3 solution) and at pH 7 (0.5 mol L^{-1} K_2SO_4 + 100 mmol L^{-1} KNO_3 solution).

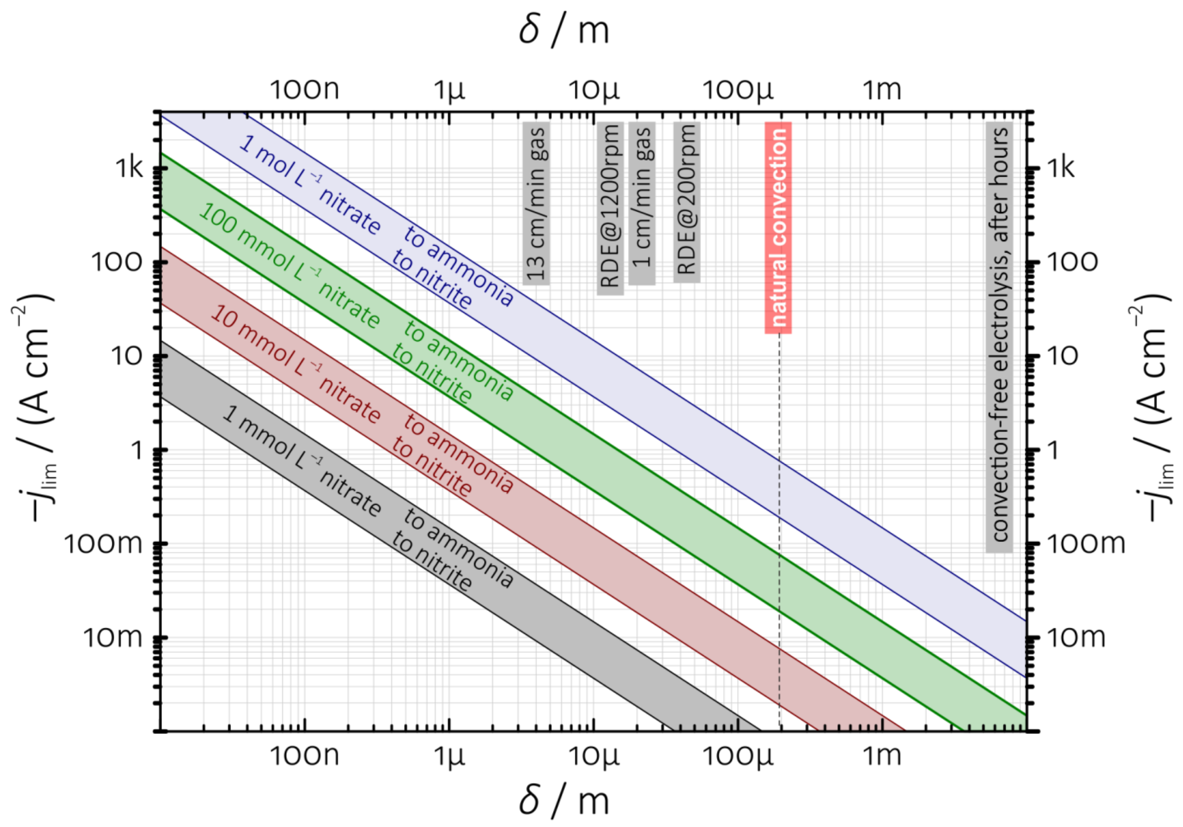

Figure S17. Estimated limiting current densities of nitrate reduction (from solutions of different nitrate concentration, yielding either ammonia or nitrite) as a function of the diffusion layer thickness (δ). Some commonly applied modes of "stirring" (e.g., rotating disc electrode (RDE) configuration given in rpm units and hydrogen gas evolution given in units of cm min^{-1}), and the thus established diffusion layer thicknesses are also shown.

From Fick's first law follows that in case of a stationary diffusion layer thickness δ, the limiting current density can be calculated as

$$j_{\mathrm{lim}} = nFD\frac{c_\infty}{\delta} \qquad (1)$$

where n denotes the number of electrons taking part in the reaction (n = 8 for nitrate→ammonia and n = 2 for nitrate→nitrite reduction); F = 96485.3 C mol^{-1} is Faraday's constant and $D = 1.902 \cdot 10^{-5}$ cm^2 s^{-1} is the (approximate) diffusion coefficient[1] of nitrate ions. The relationship between the achievable (limiting) current density and the diffusion layer thickness is shown in Figure S17 for different nitrate concentrations and reaction routes (nitrite or ammonia formation). In general, the diffusion layer thickness δ can be decreased (the limiting current increased) by the application of agitation (stirring). Natural convection, that occurs by a slow motion of electrolyte caused by electrolysis-induced density gradients, usually establishes a few hundreds of micrometers thick diffusion layers. By means of explicit agitation (e.g., by rotating the electrode), δ can be decreased to the range of tens of micrometers, while intensive gas formation can result in only a few micrometers thick diffusion layers on planar electrodes. For porous surfaces with considerable surface roughness,

under given stirring conditions, the achievable current density can, according to the literature, increase about 2-fold compared to what is shown in Figure S17.[2] According to Ibl and Venczel,[3,4,5] the continuous release **of H_2 bubbles** of an average radius *r* from the electrode surface could establish a diffusion layer thickness

$$\delta = \sqrt{\frac{\pi D r}{6 v_g}(1-\tau)}, \qquad (2)$$

where *D* denotes the diffusion coefficient of the reacting species (in our case, of nitrate ions), τ is the relative coverage of the surface by gas bubbles, and v_g is a velocity term gained by normalizing the (volumetric) rate of gas evolution to the surface area of the electrode.

The experimentally observed limiting currents for “non-stirred” (natural convection only case) and gas evolution conditions presented in Figure 5 and 7 are well within the expected range indicated in Figure S17.

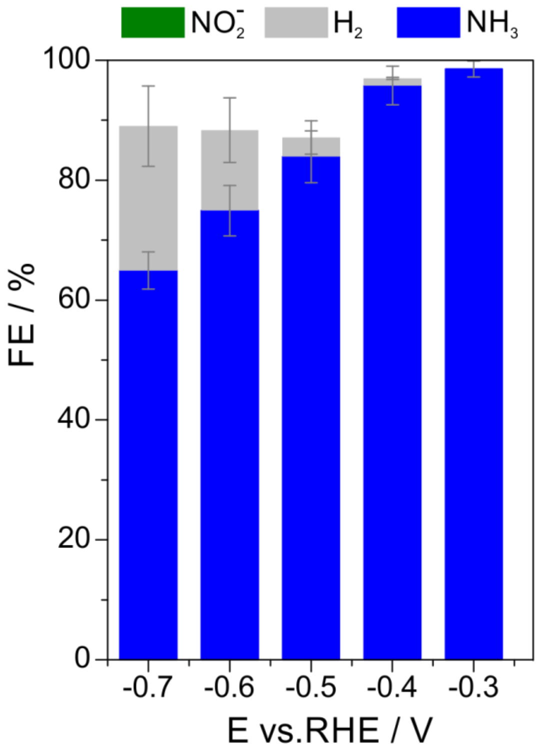

Figure S18. Product distribution of 30 min. electrolyses carried out at pH 14 (1 mol L^{-1} KOH) in 100 mmol L^{-1} KNO_3 electrolyte solution. The product analysis was extended towards online gas-chromatography exemplarily demonstrating that hydrogen is indeed the prevalent by-product of the electrolyses performed at potentials more negative than –0.4 V vs. RHE. The product distribution was derived from averaging three individual electrolyses. Note that for more cathodic electrolysis potentials the FE values do not sum up in all cases to 100%. This is most likely due to a partial loss of formed ammonia by diffusion into the anolyte (see Figure S21). The efficiencies for ammonia production may be in reality even higher than displayed in the FE plots. One important finding is that the anion exchange membrane is permeable for ammonia/ammonium (see Figure S21).

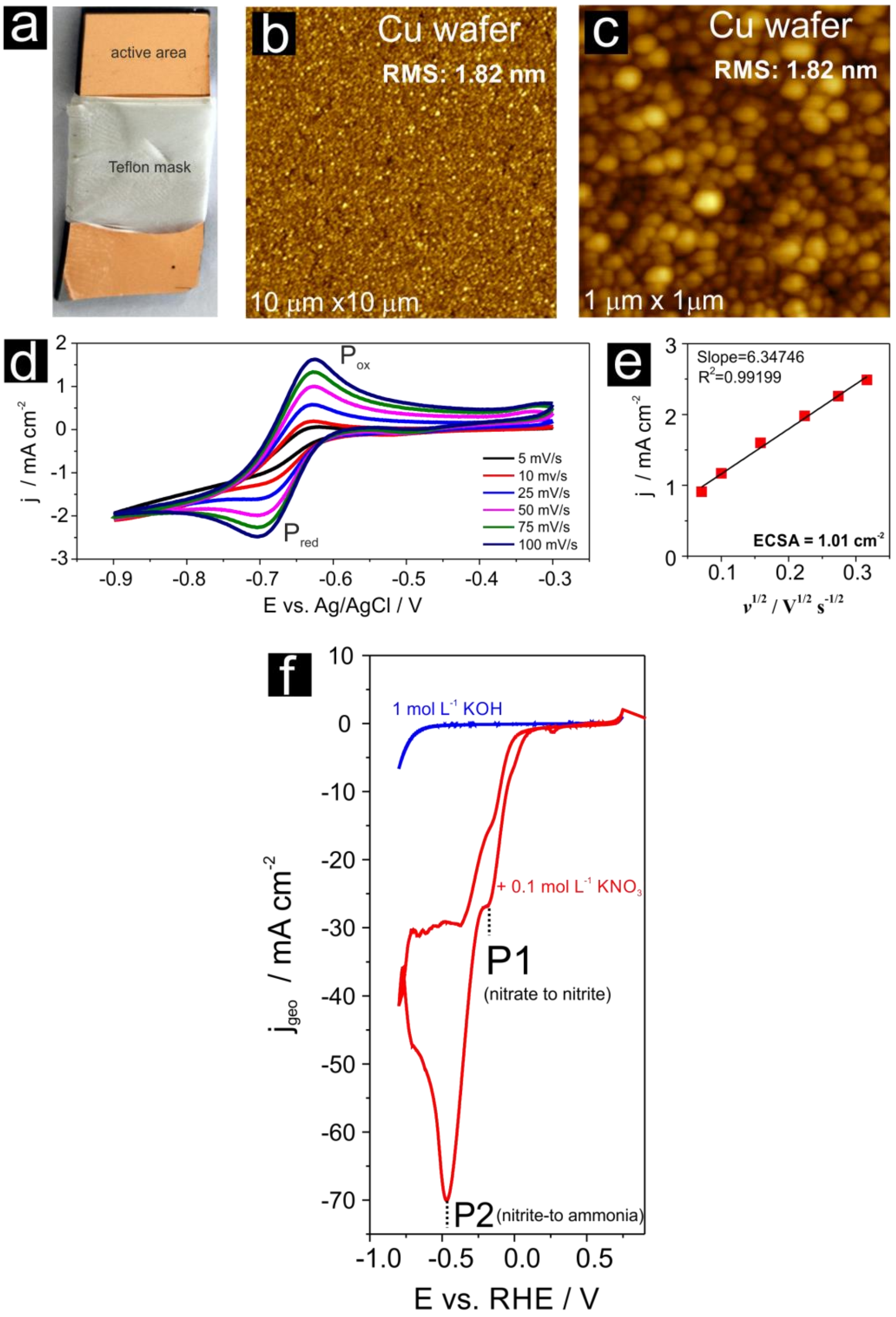

Figure S19. a) Optical micrograph showing the Cu wafer coupon used for the reference electrolysis experiment presented in Figure S17. A geometric surface area of 1 cm^2 is realized by masking the wafer coupon with insulating Teflon tape. b-c) Scanning force microscopic inspection of the Cu wafer coupon sample. d-e) Voltammetric data (viologen method, see Experimental section) used for the ESCA determination. f) Voltammetric data demonstrating the nitrate reduction on the Cu wafer coupon sample. The potential sweep rate was 25 mV s^{-1}.

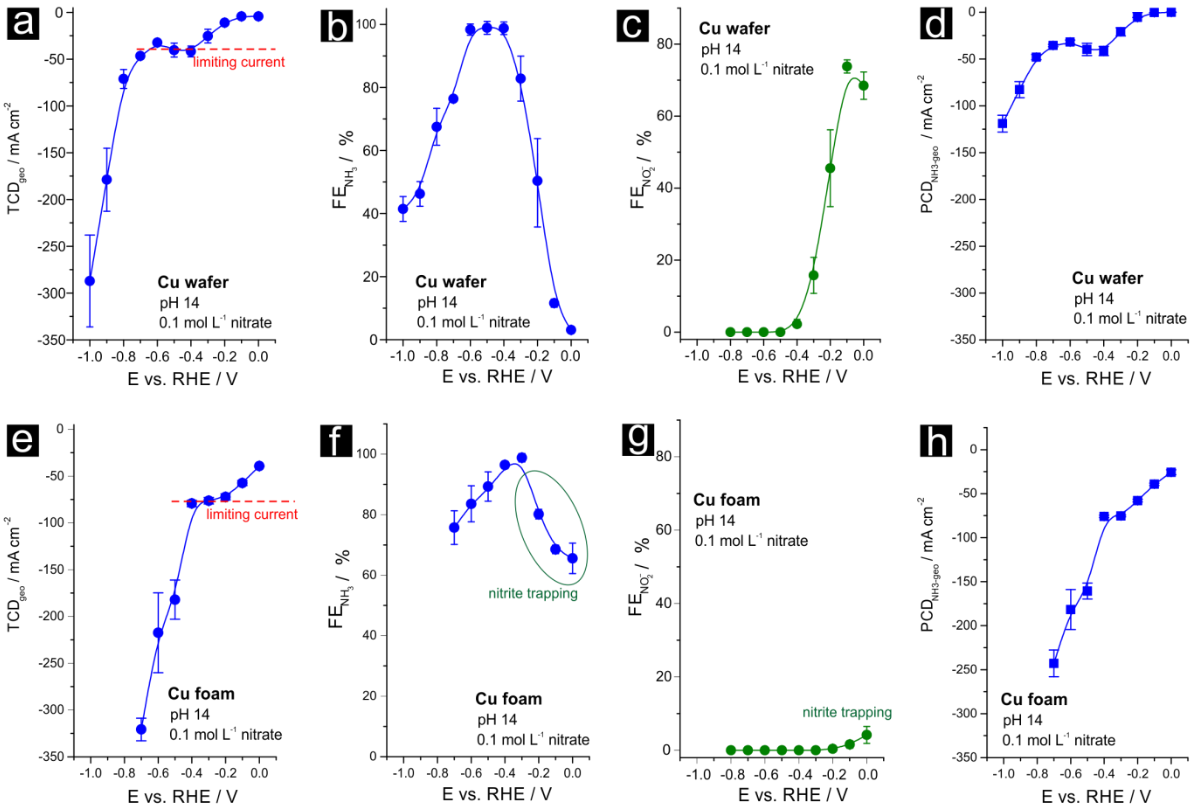

Figure S20. a-d) Electrolysis data (30 minutes electrolysis duration) obtained for a planar Cu wafer coupon sample (see Figure S19). e-f) Electrolysis data (30 minutes electrolysis duration) obtained for the Cu-foam(30 s)@mesh sample.

The comparison confirms the effective trapping and further reaction of nitrite into ammonia in case of the Cu foam catalyst (see Figure S20c and Figure S20g). By contrast to that, nitrite intermediates are readily released into the electrolyte in case of the ideally planar Cu wafer coupon sample. Of note is that these trapping effects are only effective under “competitive” experimental conditions where both products, ammonia and nitrite, form on the Cu catalyst and nitrite can in principle be released into the electrolyte (e.g., at pH 14 and 0.1 mol L^{-1} nitrate concentrations at potentials above –0.4 V vs. RHE). At more cathodic electrolysis potentials, however, adsorbed nitrite directly reacts further into ammonia. Trapping effects seem to be of less importance at these electrolysis conditions. In either case (Cu foam, wafer coupon) no nitrate could be detected as a by-product of the nitrate reduction at potentials more negative than –0.4 V vs. RHE.

A comparison further confirms the appearance of pronounced limiting currents (plateaus in the TCD_{geo} *vs.* E plots) for the nitrate reduction in both cases before the onset of the HER. The limiting current in case of the porous Cu foam is higher by a factor of ca. 2 compared to the planar Cu wafer coupon (see discussion of Figure S17, reference 2). The observed “boost” of the ammonia partial current density sets in at less cathodic potentials in the case of the Cu foam catalyst and is much more pronounced than in case of the planar Cu wafer coupon sample (see Figure S20d and Figure S20h). This observation can be rationalized, at least partly, by an ECSA which is higher by a factor of ca. 7 in case of the porous Cu foam (30 s deposition, see table S1) when compared to the planar wafer coupon surface (ECSA ca. 1 cm^2).

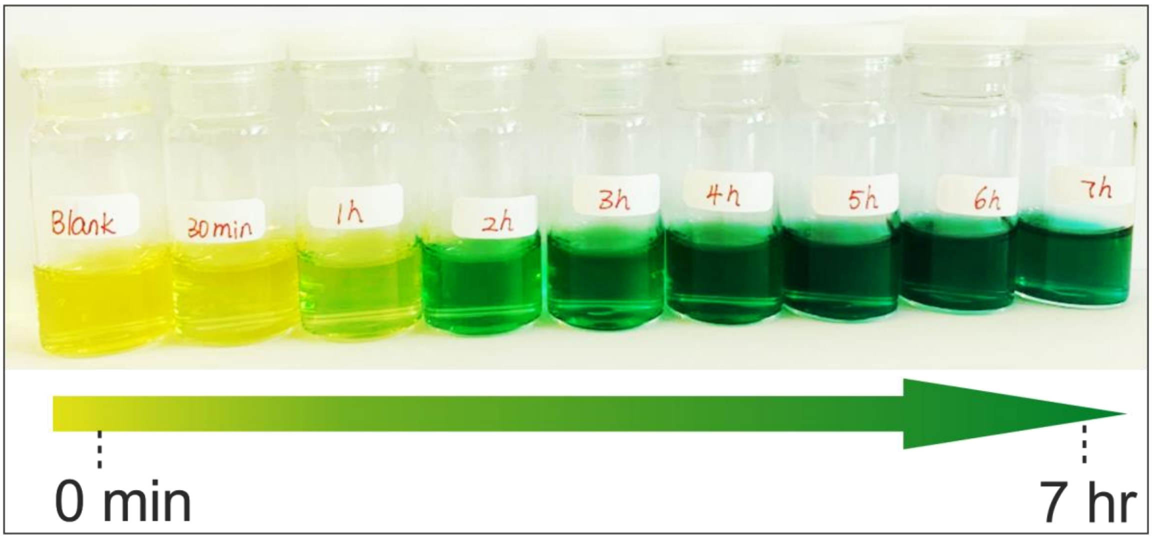

Figure S21. Time dependent accumulation of ammonia in the anolyte evidenced by the indophenol analysis approach. Samples were taken from the anolyte as a function of the electrolysis time during nitrate reduction at –0.7 V vs. RHE at pH 14 and 100 mmol L^{-1} KNO_3 electrolyte solution. A precise quantification through UV-vis was not carried out in this case due to expected partial re-oxidation of ammonia at the anode. These experiments, however, clearly demonstrate that ammonia can pass the anion exchange membrane. The FE values for ammonia production derived from the analysis of the catholyte might be in reality even higher in particular following extended electrolyses at higher reaction rates.

Deposition time / s	Foam thickness / µm	Mass loading / mg cm^{-2}	ECSA / cm^2
0	-	-	1.10±0.16
5	27.5 ± 3.53	1.7 ± 0.085	2.07 ± 0.10
10	45 ± 7.07	4.2 ± 0.21	2.93 ± 0.14
20	65 ± 7.07	8.8 ± 0.44	5.26 ± 0.26
30	85 ± 7.07	13.7 ± 0.68	7.35 ± 0.36
40	92.5 ± 10.6	18.4 ± 0.92	10.17 ± 0.50
60	150 ± 24.04	28.5 ± 1.42	12.55 ± 0.62

Table S1. Characteristics of the Cu-foam@mesh catalysts depending on the Cu deposition time. The data presented herein correspond to Figure 3 and Figure S4.

Deposition time/s	–0.1 V vs. RHE			–0.3 V vs. RHE		
	TCD_{geo} / mA cm^{-2}	FE_{NH3} / %	PCD_{NH3} / mA cm^{-2}	TCD_{geo} / mA cm^{-2}	FE_{NH3} / %	PCD_{NH3} / mA cm^{-2}
5	–49.9 ± 2.2	38.3 ± 1.4	–19.1 ± 0.7	–70.1 ± 1.4	63.3 ± 5.0	–39.8 ± 2.0
10	–59.2 ± 2.2	46.6 ± 2.3	–27.6 ± 0.4	–72.3 ± 1.1	72.1 ± 2.0	–52.1 ± 0.7
20	–61.8 ± 0.5	65.6 ± 3.6	–40.5 ± 0.4	–74.5 ± 4.5	84.1 ± 2.1	–62.7 ± 2.2
30	–63.8 ± 2.5	68.9 ± 12.5	–43.7 ± 9.5	–76.2 ± 1.6	98.5 ± 1.4	–75.0 ± 0.6
40	–65.9 ± 0.6	82.6 ± 3.9	–52.8 ± 2.0	–80.4 ± 4.9	95.8 ± 3.0	–77.0 ± 2.2
60	–73.5 ± 2.0	51.9 ± 2.9	–38.1 ± 0.9	–84.0 ± 3.5	78.5 ± 4.2	–66.0 ± 0.8

Table S2. Total current density (TCD_{geo}), faradaic efficiency (FE) and partial current density (PCD) for ammonia production derived for various Cu-foam@mesh catalysts after 30 min potentiostatic NO_3^-RR in 100 mmol L^{-1} KNO_3 electrolyte solution at electrolysis potentials of –0.1 and –0.3 V vs. RHE. The presented data correspond to Figure 5a, 5b, and 5c. Note that the total and the partial current density were normalized to the geometric surface area. The presented numerical values were derived from an averaging of three individual experiments.

Potential / V	Cu mesh			Cu-foam(30 s)@mesh		
	TCD_{geo} / mA cm^{-2}	FE_{NH3} / %	PCD_{NH3} / mA cm^{-2}	TCD_{geo} / mA cm^{-2}	FE_{NH3} / %	PCD_{NH3} / mA cm^{-2}
–0.1	–3.2 ± 1.4	13.9 ± 2.5	–0.4 ± 0.2	–63.8 ± 2.5	68.9 ± 12.5	–43.7 ± 9.5
–0.2	–5.9 ± 0.8	25.6 ± 3.2	–1.5 ± 0.0	–72.2 ± 3.3	80.2 ± 1.6	–57.9 ± 1.5
–0.3	–10.1 ± 3.6	57.8 ± 2.4	–5.8 ± 2.0	–76.2 ± 1.6	98.8 ± 1.4	–75.3 ± 1.0
–0.4	–31.5 ± 4.4	74.9 ± 2.0	–23.6 ± 2.7	–79.2 ± 2.4	95.8 ± 1.2	–75.9 ± 1.3
–0.5	–42.7 ± 4.7	74.7 ± 1.6	–31.9 ± 2.8	-182.0 ± 21.0	88.3 ± 4.8	-160.7 ± 9.7–
–0.6	–73.7 ± 3.2	57.2 ± 1.2	–42.2 ± 0.9	-217.4 ±42.6	83.6 ± 6.0	-181.7 ± 22.7
–0.7	–170.6 ± 4.1	41.8 ± 1.3	–71.3 ± 0.5	-320.7 ± 12.0	75.7 ± 5.6	-242.9 ± 15.2

Table S3. Potential dependent Faradaic efficiency data for ammonia (main NO_3^-RR product) production. The electrolysis data were obtained after 30 min of potentiostatic electrolysis at pH 14 in 100 mmol L^{-1} KNO_3 electrolyte solution. For each electrolysis a newly prepared catalyst was used. The table displays the averaged FE values from three individual electrolyses per applied potential. The presented data correspond to Figure 5d, 5e, and 5f.

Potential / V	10 mmol L^{-1}		100 mmol L^{-1}		500 mmol L^{-1}	
	FE_{NH3}/ %	$FE_{nitrite}$ / %	FE_{NH3} /%	$FE_{nitrite}$ / %	FE_{NH3}/ %	$FE_{nitrite}$ / %
0	58.4 ± 0.6	8.6 ± 8.6	65.6 ± 5.0	4.2 ± 2.3	34.8 ± 4.5	59.1 ± 3.4
–0.1	97.0 ± 1.9	1.3 ± 0.2	68.9 ± 12.5	1.6 ± 1.0	66.5 ± 3.2	29.5 ± 4.5
–0.2	100.0 ± 3.35	0	80.2 ± 1.6	0.4 ± 0.2	75.5 ± 3.2	14.6 ± 3.5
–0.3	69.4 ± 5.7	0	98.8 ± 1.4	0	84.6 ± 3.3	5.9 ± 2.1
–0.4	60.2 ± 3.3	0	95.8 ± 1.2	0	90.5 ± 5.3	2.0 ± 1.2
–0.5	46.6 ± 5.2	0	88.3 ± 4.8	0	95.2 ± 3.5	1.7 ± 2.3
–0.6	26.9 ± 3.2	0	83.6 ± 6.0	0	78.3 ± 3.5	1.6 ± 2.2
–0.7	21.5 ± 1.3	0	75.7 ± 5.6	0	65.34 ± 2.5	1.5 ± 1.3

Table S4. Nitrate concentration (10 mmol L^{-1}, 100 mmol L^{-1}, and 500 mmol L^{-1}) and potential dependent Faradaic efficiency data for ammonia (main NO_3^-RR product) and nitrite (parasitic NO_3^-RR by-product). The electrolysis data were obtained after 30 min of potentiostatic electrolysis at **pH 14**. For each electrolysis a newly prepared catalyst was used. The table displays the averaged FE values from three individual electrolyses per applied potential. The presented data correspond to Figure 7a.

Potential / V	10 mmol L^{-1}			100 mmol L^{-1}			500 mmol L^{-1}		
	S_{NH3} / %	$S_{nitrite}$ / %	S_{others} / %	S_{NH3} / %	$S_{nitrite}$ / %	S_{others} / %	S_{NH3} / %	$S_{nitrite}$ / %	S_{others} / %
0	58.3 ± 2.3	37.5 ±4.1	4.1 ± 0.7	55.2 ± 4.6	32.7 ± 3.6	12.1 ± 2.1	13.5± 1.2	76.3± 3.2	10.3 ± 2.1
–0.1	94.8 ± 0.5	5.1 ± 0.0	0.03 ± 0.01	75.5 ± 3.3	8.8 ± 2.4	15.7 ± 1.3	34.6 ± 2.3	54.8 ± 2.6	10.7 ± 3.2
–0.2	99.9 ± 0.4	0	0.15 ± 0.01	88.9 ± 2.1	1.7 ± 2.5	9.3 ± 1.5	52.3 ± 1.3	35.4 ± 1.3	12.3 ± 1.5
–0.3	99.3 ± 0.6	0	0.7 ± 0.5	99.0 ± 0.4	0	0.4 ± 0.1	70.7 ± 2.4	19.2 ± 1.6	10.1 ± 1.2
–0.4	98.1 ± 0.9	0	1.9 ± 1.2	99.5 ± 0.3	0	0.56 ± 0.1	82.6 ± 4.9	15.9 ± 1.8	1.5 ± 0.9
–0.5	98.4 ± 1.1	0	1.6 ± 1.0	98.9 ± 0.6	0	1.2 ± 0.4	93.3 ± 2.4	5.5 ± 1.3	1.3 ± 0.8
–0.6	98.1 ± 1.0	0	1.9 ± 1.0	98.6 ± 1.2	0	1.4 ± 0.3	92.7 ± 2.4	3.6 ± 2.5	3.7± 0.7
–0.7	98.0 ± 0.9	0	1.9 ± 0.9	97.9 ± 1.0	0	2.1 ± 0.4	91.5 ± 2.2	2.5 ± 2.1	6.1± 0.4

Table S5. Nitrogen selectivity (S_{NH3}, $S_{nitrite}$, S_{others}) data for the potential and nitrate dependent electrolyses experiments at **pH 14**. The data displayed in the table correspond to Figure 7b.

Potential / V	10 mmol L^{-1}		100 mmol L^{-1}		500 mmol L^{-1}	
	PCD_{NH3} / mA cm^{-2}	$PCD_{nitrite}$ / mA cm^{-2}	PCD_{NH3} / mA cm^{-2}	$PCD_{nitrite}$ / mA cm^{-2}	PCD_{NH3} / mA cm^{-2}	$PCD_{nitrite}$ / mA cm^{-2}
0	–2.2 ± 0.8	–0.3 ± 1.2	–25.7 ± 2.7	–4.8 ± 0.8	–17.5 ± 2.4	–29.7 ± 1.8
–0.1	–4.0 ± 1.1	–0.1 ± 0.3	–43.7 ± 9.5	–1.0 ± 0.6	–141.0 ± 9.6	–62.5 ± 10.3
–0.2	–4.5 ± 1.7	0	–57.9 ± 1.5	–0.3 ± 0.1	–203.0 ± 45.1	–39.2 ± 45.3
–0.3	–3.7 ± 3.2	0	–75.3 ± 1.0	0	–420.6 ± 4.4	–29.1 ± 3.8
–0.4	–8.00 ± 3.1	0	–75.9 ± 1.3	0	–524.6 ± 49.7	–11.3 ± 47.6
–0.5	–15.4 ± 3.7	0	-160.7 ± 9.7–	0	–716.6 ± 17.3	–12.6 ± 16.7
–0.6	–12.6 ± 7.3	0	-181.7 ± 22.7	0	–952.6 ± 19.7	–19.1 ± 19.1
–0.7	–18.5 ± 4.2	0	-242.9 ± 15.2	0	–1045.4 ± 34.4	–26.1 ± 14.0

Table S6. Nitrate concentration (10 mmol L^{-1}, 100 mmol L^{-1}, and 500 mmol L^{-1}) and potential dependent partial current densities (PCDs) for ammonia (main NO_3^-RR product) and nitrite (parasitic NO_3^-RR by-product). The electrolysis data was obtained after 30 min of potentiostatic electrolysis at **pH 14**. The presented data correspond to Figure 7c. The current densities were **normalized to the geometric surface area**.

Potential / V	10 mmol L^{-1}		100 mmol L^{-1}		500 mmol L^{-1}	
	PCD_{NH3} / mA cm^{-2}	$PCD_{nitrite}$ / mA cm^{-2}	PCD_{NH3} / mA cm^{-2}	$PCD_{nitrite}$ / mA cm^{-2}	PCD_{NH3} / mA cm^{-2}	$PCD_{nitrite}$ / mA cm^{-2}
0	–0.3 ± 0.1	–0.04 ± 0.02	–3.5 ± 0.4	–0.7 ± 0.1	–2.4 ± 0.3	–4.0 ± 0.3
–0.1	–0.6 ± 0.2	–0.01 ± 0.03	–5.9 ± 1.3	–0.2 ± 0.2	–19.2 ± 1.3	–8.5 ± 1.4
–0.2	–0.6 ± 0.2	0	–8.0 ± 0.2	–0.1 ± 0.1	–27.6 ± 6.1	–5.3 ± 6.2
–0.3	–0.5 ± 0.4	0	–10.2 ± 1.0	0	–57.2 ± 0.6	–4.0 ± 0.5
–0.4	–1.1 ± 0.4	0	–10.3 ± 0.6	0	–71.4 ± 6.8	–1.5 ± 6.5
–0.5	–2.1 ± 0.5	0	–21.9 ± 4.1	0	–97.5 ± 2.4	–1.7 ± 2.3
–0.6	–1.8 ± 1.0	0	–24.7 ± 9.7	0	–129.6 ± 2.7	–2.6 ± 2.6
–0.7	–2.5 ± 0.6	0	–33.0 ± 3.7	0	–142. 2 ± 2.9	–3.5 ± 3.3

Table S7. Nitrate concentration (10 mmol L^{-1}, 100 mmol L^{-1}, and 500 mmol L^{-1}) and potential dependent partial current densities (PCDs) for ammonia (main NO_3^-RR product) and nitrite (parasitic NO_3^-RR by-product). The electrolysis data was obtained after 30 min of potentiostatic electrolysis at **pH 14**. The current densities presented from Table S6 were **normalized (herein) to the electrochemically active surface area (ECSA)**.

Potential / V	10 mmol L^{-1}		100 mmol L^{-1}		500 mmol L^{-1}	
	FE_{NH3}/ %	$FE_{nitrite}$ / %	FE_{NH3} /%	$FE_{nitrite}$ / %	FE_{NH3}/ %	$FE_{nitrite}$ / %
–0.1	4.7 ± 1.3	29.1 ± 2.9	1.8 ± 0.1	69.5 ± 0.7	1.1 ± 0.5	81.3 ± 3.4
–0.2	29.1 ± 2.1	22.1 ± 4.2	12.1 ± 4.4	65.2 ± 5.1	1.6 ± 0.8	80.2 ± 7.8
–0.3	89.3 ± 3.6	5.9 ± 0.9	37.1 ± 0.5	57.3 ± 3.2	5.5 ± 0.7	78.6 ± 4.5
–0.4	93.3 ± 1.9	2.1 ± 0.1	84.1 ± 3.2	11.9 ± 3.1	24.2 ± 1.3	57.1 ± 4.5
–0.5	100.0 ± 2.4	-	92.3 ± 3.2	3.9 ± 2.3	50.8 ± 3.3	35.3 ± 4.3
–0.6	95.3 ± 2.1	-	94.3 ± 4.2	-	64.8 ± 2.3	18.7 ± 1.2
–0.7	70.4 ± 4.2	-	99.0 ± 2.1	-	77.8 ± 1.6	12.5 ± 1.6
–0.8	62.9 ± 3.2	-	95.8 ± 3.2	-	84.4 ± 3.3	7.6 ± 1.5
–0.9	49.1 ± 2.4	-	87.0 ± 4.7	-	88.1 ± 3.2	4.4 ± 0.6
–1.0	38.0 ± 2.3	-	76.7 ± 4.7	-	80.9 ± 2.3	2.9 ± 0.9

Table S8. Nitrate concentration (10 mmol L^{-1}, 100 mmol L^{-1}, and 500 mmol L^{-1}) and potential dependent Faradaic efficiency data for ammonia (main NO_3^-RR product) and nitrite (parasitic NO_3^-RR by-product). The electrolysis data was obtained after 30 min of potentiostatic electrolysis at **pH 7**. For each electrolysis a newly prepared catalyst was used. The table displays the averaged FE values from three individual electrolyses per applied potential. The presented data correspond to Figure 7d.

Potential / V	10 mmol L^{-1}			100 mmol L^{-1}			500 mmol L^{-1}		
	S_{NH3} / %	$S_{nitrite}$ / %	S_{others} / %	S_{NH3} / %	$S_{nitrite}$ / %	S_{others} / %	S_{NH3} / %	$S_{nitrite}$ / %	S_{others} / %
–0.1	3.1 ± 0.4	93.0 ± 0.6	4.0 ± 4.0	0.6 ± 1.2	81.3 ± 3.4	18.1 ± 2.3	0.6 ± 0.5	86.5 ± 2.3	12.9 ± 2.1
–0.2	27.5 ± 3.6	70.3 ± 0.7	2.2 ± 2.2	10.1 ± 2.3	71.8 ± 2.4	18.0 ± 3.3	0.6 ± 0.5	87.9 ± 2.5	11.6 ± 1.8
–0.3	76.5 ± 1.2	21.0 ± 0.4	2.4 ± 2.4	22.9 ± 2.1	65.2 ± 1.5	11.9 ± 2.6	1.9 ± 0.8	87.7 ± 3.2	10.4 ± 1.8
–0.4	90.3 ± 0.3	7.6 ± 0.8	2.2 ± 2.2	63.5 ± 3.5	35.2 ± 2.1	1.8 ± 1.3	8.6 ± 1.2	84.5 ± 3.5	6.9 ± 1.5
–0.5	100.0 ± 2.5	0	0.01 ± 0.0	83.6 ± 4.5	14.0 ± 2.2	2.4 ± 3.2	23.2 ± 1.5	67.3 ± 2.3	9.6 ± 1.2
–0.6	99.0 ± 1.2	0	1.0 ± 1.0	93.5 ± 1.5	5.0 ± 1.0	1.5 ± 1.3	40.1 ± 2.1	49.7 ± 1.3	10.1 ± 1.3
–0.7	95.1 ± 2.1	0	4.9 ± 4.9	99.0 ± 2.1	0	0.9 ± 1.4	53.5 ± 2.3	34.8 ± 1.3	11.7 ± 0.9
–0.8	93.6 ± 3.1	0	6.4 ± 6.4	98.3 ± 2.3	0	1.7 ± 1.2	61.1 ± 3.2	28.7 ± 2.1	10.2 ± 1.2
–0.9	90.5 ± 1.3	0	9.5 ± 9.5	98.7 ± 1.6	0	1.3 ± 1.2	80.6 ± 3.1	18.2 ± 1.2	1.2 ± 0.8
-1.0	90.2 ± 1.1	0	9.7 ± 9.7	94.7 ± 1.3	0	4.8 ± 1.8	85.3 ± 3.8	10.6 ± 1.5	4.1 ± 1.8

Table S9. Nitrogen selectivity (S_{NH3}, $S_{nitrite}$, S_{others}) data for the potential and nitrate dependent electrolyses experiments at **pH 7**. The data displayed in the table correspond to Figure 7e.

Potential / V	10 mmol L^{-1}		100 mmol L^{-1}		500 mmol L^{-1}	
	PCD_{NH3} / mA cm^{-2}	$PCD_{nitrite}$ / mA cm^{-2}	PCD_{NH3} / mA cm^{-2}	$PCD_{nitrite}$ / mA cm^{-2}	PCD_{NH3} / mA cm^{-2}	$PCD_{nitrite}$ / mA cm^{-2}
–0.1	–0.1 ± 0.7	–0.7 ± 1.5	–0.1 ± 0.1	–3.8 ± 0.4	–0.1 ± 0.3	–9.4 ± 1.7
–0.2	–0.8 ± 1.5	–0.6 ± 2.6	–1.2 ± 2.9	–6.2 ± 3.3	–0.5 ± 2.2	–24.6 ± 5.8
–0.3	–2.8 ± 2.0	–0.2 ± 0.7	–4.5 ± 0.4	–7.0 ± 1.8	–2.4 ± 2.2	–34.6 ± 4.1
–0.4	–3.6 ± 1.0	–0.1 ± 0.1	–21.1 ± 2.8	–3.0 ± 2.8	–17.0 ± 0.8	–40.1 ± 2.4
–0.5	–3.6 ± 1.4	0	–41.1 ± 2.5	–1.8 ± 2.0	–63.7 ± 4.0	–44.1 ± 4.5
–0.6	–3.8 ± 1.2	0	–49.8 ± 5.9	0	–125.0 ± 6.9	–36.2 ± 6.3
–0.7	–5.1 ± 2.3	0	–63.1 ± 3.5	0	–214.4 ± 2.6	–34.4 ± 2.5
–0.8	–11.6 ± 1.9	0	–74.9 ± 6.1	0	–256.5 ± 4.9	–23.0 ± 4.1
–0.9	–17.5 ± 1.8	0	–95.0 ± 4.3	0	–331.0 ± 4.3	–16.6 ± 3.0
–1.0	–22.8 ± 6.2	0	–120.1 ± 7.8	0	–366.7 ± 11.6	–13.0 ± 10.6

Table S10. Nitrate concentration (10 mmol L^{-1}, 100 mmol L^{-1}, and 500 mmol L^{-1}) and potential dependent partial current densities (PCDs) for ammonia (main NO_3^-RR product) and nitrite (parasitic NO_3^-RR by-product). The electrolysis data was obtained after 30 min of potentiostatic electrolysis at **pH 7**. The presented data correspond to Figure 7f. The current densities were **normalized to the geometric surface area**.

Potential / V	10 mmol L^{-1}		100 mmol L^{-1}		500 mmol L^{-1}	
	PCD_{NH3} / mA cm^{-2}	$PCD_{nitrite}$ / mA cm^{-2}	PCD_{NH3} / mA cm^{-2}	$PCD_{nitrite}$ / mA cm^{-2}	PCD_{NH3} / mA cm^{-2}	$PCD_{nitrite}$ / mA cm^{-2}
–0.1	–0.02 ± 0.01	–0.1 ± 0.2	–0.01 ± 0.02	–0.5 ± 0.1	–0.02 ± 0.04	–1.3 ± 0.23
–0.2	–0.1 ± 0.2	–0.1 ± 0.4	–0.2 ± 0.4	–0.8 ± 0.5	–0.07 ± 0.30	–3.4 ± 0.8
–0.3	-0.4 ± 0.3	–0.03 ± 0.10	–0.6 ± 0.1	–1.0 ± 0.2	–0.3 ± 0.3	–4.7 ± 0.6
–0.4	–0.5 ± 0.1	–0.01 ± 0.01	–2.9 ± 0.4	–0.4 ± 0.4	–2.3 ± 0.1	–5.5 ± 0.3
–0.5	–0.5 ± 0.2	0	–5.6 ± 0.3	–0.2 ± 0.3	–8.7 ± 0.5	–6.0 ± 0.6
–0.6	–0.5 ± 0.2	0	–6.8 ± 0.8	0	–17.0 ± 0.9	–4.9 ± 0.9
–0.7	–0.7 ± 0.3	0	-8.6 ± 0.5	0	–29.2 ± 0.4	–4.7 ± 0.3
–0.8	-–1.6 ± 0.3	0	–10.2 ± 0.8	0	–34.9 ± 0.7	–3.1 ± 0.6
–0.9	–2.8 ± 0.2	0	–12.9 ± 0.6	0	–45.0 ± 0.6	–2.3 ± 0.4
–1.0	–3.1 ± 0.8	0	–16.3 ± 1.1	0	–49.9 ± 1.6	–1.8 ± 1.4

Table S11. Nitrate concentration (10 mmol L^{-1}, 100 mmol L^{-1}, and 500 mmol L^{-1}) and potential dependent partial current densities (PCDs) for ammonia (main NO_3^-RR product) and nitrite (parasitic NO_3^-RR by-product). The electrolysis data was obtained after 30 min of potentiostatic electrolysis at **pH 7**. The current densities from Table S10 were **normalized (herein) to the electrochemically active surface area (ECSA)**.

Time / h	pH 14 –0.3 V vs. RHE				pH 7 –0.3 V vs. RHE			
	FE_{NH3} / %	PCD_{NH3} / mA cm^{-2}	$FE_{nitrite}$ / %	$PCD_{nitrite}$ / mA cm^{-2}	FE_{NH3} / %	PCD_{NH3} / mA cm^{-2}	$FE_{nitrite}$ / %	$PCD_{nitrite}$ / mA cm^{-2}
**0.5**	**99.1**	**–115.6**	**0**	**0**	**38.7**	**–7.0**	**57.4**	**–10.4**
2.0	90.9	–71.9	0.3	–0.3	31.9	–5.1	64.6	–10.2
5.0	85.5	–52.8	0.2	–0.1	26.8	–3.6	66.2	–9.0
7.0	79.1	–43.4	0.2	–0.1	25.1	–3.1	62.7	–7.7
**7.5**	**101.2**	**–102.2**	**0**	**0**	**41.2**	**–6.6**	**51.2**	**–8.3**
9.0	88.0	–73.9	0.5	–0.4	34.5	–5.0	59.1	–8.6
12.0	82.1	–55.3	0.3	–0.2	26.6	–3.7	60.8	–8.6
14.0	77.3	–46.2	0.2	–0.1	24.4	–3.2	58.9	–7.8
**14.5**	**97.5**	**–86.3**	**0.6**	**–0.5**	**47.5**	**–10.9**	**42.3**	**–9.7**
16.0	79.4	–65.2	0.4	–0.4	40.3	—9.0	43.5	–9.7
19.0	75.7	–51.5	0.4	–0.3	31.5	–6.0	44.9	–8.5
21.0	71.3	–44.3	0.4	–0.2	27.7	–4.7	44.9	–7.7
**21.5**	**95.6**	**–86.1**	**1.5**	**–1.4**	**54.5**	**–13.9**	**41.7**	**–10.6**
23.0	74.6	–61.0	0.8	–0.7	44.9	–9.2	43.7	–8.9
26.0	70.4	–46.1	0.6	–0.4	33.9	–6.1	43.8	–7.8
28.0	67.3	–40.0	0.4	–0.2	30.3	–4.9	47.2	–7.6
**28.5**	**94.4**	**–88.4**	**1.9**	**–1.8**	**54.3**	**–15.7**	**36.4**	**–10.5**
30.0	72.3	–57.7	0.8	–0.6	41.1	–10.3	40.4	–10.1
33.0	68.3	–46.5	0.6	–0.4	30.7	–6.1	49.9	–9.9
35.0	66.0	–42.8	0.4	–0.3	26.2	–4.8	51.4	–9.4
**35.5**	**91.2**	**–64.4**	**2.5**	**–1.8**	**55.8**	**–16.5**	**36.6**	**–10.8**
37.0	71.3	–48.7	1.1	–0.8	40.3	–10.4	40.4	–10.4
40.0	66.1	–37.6	0.5	–0.3	31.6	–6.8	47.8	–10.3
42.0	64.2	–33.7	0.4	–0.2	27.4	–5.2	48.7	–9.3

Table S12. Time dependent electrolysis data corresponding to Figure 8 a and c (potentiostatic electrolyses at –0.3 V vs. RHE). The current densities were normalized to the geometric surface area. The "initial" values (determined 30 min after start of the respective continuous electrolysis) are highlighted in red.

Time / h	pH 14 –0.7 V vs. RHE				pH 7 –0.7 V vs. RHE			
	FE_{NH3} / %	PCD_{NH3} / mA cm^{-2}	$FE_{nitrite}$ / %	$PCD_{nitrite}$ / mA cm^{-2}	FE_{NH3} / %	PCD_{NH3} / mA cm^{-2}	$FE_{nitrite}$ / %	$PCD_{nitrite}$ / mA cm^{-2}
**0.5**	**79.9**	**–183.9**	**0**	**0**	**98.1**	**1.9**	**–78.0**	**–1.5**
2.0	73.5	–166.3	0	0	91.1	0.9	–63.1	–0.6
5.0	54.8	–107.2	0	0	83.4	0.8	–51.9	–0.5
7.0	38.9	–71.59	0	0	79.6	0.7	–45.8	–0.4
**7.5**	**80.5**	**–178.1**	**0**	**0**	**97.9**	**2.1**	**–86.9**	**–1.9**
9.0	75.4	–158.6	0	0	90.1	0.9	–65.3	–0.6
12.0	53.4	–98.0	0	0	82.3	0.5	–49.4	–0.3
14.0	43.2	–76.1	0	0	79.0	0.3	–43.6	–0.2
**14.5**	**73.6**	**–230.0**	**0**	**0**	**95.3**	**3.7**	**–63.3**	**–.4**
16.0	60.1	–177.8	0	0	87.9	1.1	–57.4	–0.7
19.0	41.8	–105.6	0	0	84.5	0.3	–46.6	–0.2
21.0	31.8	–76.6	0	0	79.0	0.2	–40.6	–0.1
**21.5**	**69.0**	**–228.9**	**0**	**0**	**93.5**	**4.1**	**–68.6**	**–3.0**
23.0	56.1	–171.3	0	0	85.8	1.4	–62.9	–1.1
26.0	34.4	–94.4	0	0	81.5	0.8	–51.8	–0.5
28.0	24.1	–64.9	0	0	75.6	0.8	–44.2	–0.4
**28.5**	**56.6**	**–185.6**	**0**	**0**	**89.4**	**4.4**	**–79.1**	**–3.8**
30.0	49.7	–145.7	0	0	85.5	0.9	-60.8	–0.6
33.0	32.4	–85.0	0	0	79.2	0.6	-48.4	–0.4
35.0	22.6	–58.9	0	0	73.5	0.4	–42.7	–0.2
**35.5**	**55.5**	**–187.4**	**0**	**0**	**86.7**	**5.2**	**–63.9**	**–3.8**
37.0	49.4	–158.6	0	0	78.9	1.4	–53.9	–0.9
40.0	37.2	–110.3	0	0	71.2	0.7	–43.0	–0.4
42.0	27.8	–82.8	0	0	66.8	0.5	–37.6	–0.3

Table S13. Time dependent electrolysis data corresponding to Figure 8 b and d (potentiostatic electrolyses at –0.7 V vs. RHE). The current densities were normalized to the geometric surface area. The “initial” values (determined 30 min after start of the respective continuous electrolysis) are highlighted in red.

Time / h	pH 14 –0.3 V vs RHE					nitrite conc. / mmol L^{-1}		
	Cu^{2+} conc. / mg L^{-1}	Time / h	Cu^{2+} conc. / mg L^{-1}	Time / h	Cu^{2+} conc. / mg L^{-1}	Time / h	No addition of Cu^{2+}	Addition of Cu^{2+}
0.5	0.0	14.5	0.6	28.5	1.6	0.5	0 ± 0.0	0.00 ± 0.00
1.0	0.0	15.0	0.6	29.0	1.7	1	0.07 ± 0.02	0.00 ± 0.00
2.0	0.0	16.0	0.6	30.0	1.9	2	0.26 ± 0.02	0.24 ± 0.01
3.0	0.0	17.0	0.5	31.0	2.0	3	0.25 ± 0.02	0.40 ± 0.02
4.0	0.0	18.0	0.6	32.0	2.1	4	0.34 ± 0.02	0.50 ± 0.02
5.0	0.0	19.0	0.5	33.0	1.8	5	0.40 ± 0.03	0.52 ± 0.02
6.0.	0.0	20.0	0.4	34.0	1.8	6	0.45 ± 0.02	0.63 ± 0.02
7.0	0.0	21.0	0.9	35.0	2.2	7	0.48 ± 0.03	0.64 ± 0.02
7.5	0.4	21.5	1.5	35.5	8.5			
8.0	0.4	22.0	0.9	36.0	9.0			
9.0	0.4	23.0	0.8	37.0	9.4			
10.0	0.4	24.0	0.8	38.0	14.5			
11.0	0.4	25.0	0.7	39.0	15.4			
12.0	0.3	26.0	0.7	40.0	15.6			
13.0	0.3	27.0	0.6	41.0	16.6			
14.0	0.6	28.0	2.7	42.0	18.8			

Table S14. Time dependent electrolysis data corresponding to Figure 10b and c. The nitrite efficiency was monitored together with the concentration cupric ions in the electrolyte solution during NO_3 electrolysis at pH 14 and –0.3 V vs. RHE. The control experiments were performed by addition of known concentration Cu(II) ions in the electrolyte solution.

Potential / V	Cu wafer				Cu-foam(30 s)@mesh			
	TCD_{geo} / mA cm^{-2}	FE_{NH3} / %	FE_{NO2} /%	PCD_{NH3} / mA cm^{-2}	TCD_{geo} / mA cm^{-2}	FE_{NH3} / %	FE_{NO2} /%	PCD_{NH3} / mA cm^{-2}
0	-4.2±0.8	3.1±1.0	68.5±3.8	-0.1±0.02	-39.2±0.4	65.6 ± 5.0	4.2 ± 2.3	-25.7±2.7
–0.1	-4.3±0.4	11.6±1.3	73.8±1.8	-0.5±0.01	–63.8 ± 2.5	68.9 ± 12.5	1.6 ± 1	–43.7 ± 9.5
–0.2	-11.1±3.0	50.3±13.4	45.5±10.7	-5.6±0.02	–72.2 ± 3.3	80.2 ± 1.6	0.4 ± 0.2	–57.9 ± 1.5
–0.3	-25.3±7.4	82.8±7.1	15.8±5.0	-20.9±4.3	–76.2 ± 1.6	98.8 ± 1.4	0	–75.3 ± 1.0
–0.4	-41.8±5.8	99.4±2.0	2.2±1.3	-41.5±4.9	–79.2 ± 2.4	95.8 ± 1.2	0	–75.9 ± 1.3
–0.5	-40.3±7.5	98.9±2.1	0	-39.8±6.6	-182.0 ± 21.0	88.3 ± 4.8	0	-160.7 ± 9.7–
–0.6	-32.2±2.8	98.7±1.8	0	-31.8±2.2	-217.4 ±42.6	83.6 ± 6.0	0	-181.7 ± 22.7
–0.7	-46.5±2.2	76.4±0.9	0	-35.6±1.2	-320.7 ± 12.0	75.7 ± 5.6	0	-242.9 ± 15.2
-0.8	-71.1±10.2	67.5±5.8	0	-48.0±2.7	-	-	-	-
-0.9	-178.8±33.7	46.2±3.9	0	-82.7±8.6	-	-	-	-
-1.0	-287.1±49.1	41.4±4.0	0	-118.9±9.0	-	-	-	-

Table S15 Potential dependent Faradaic efficiency data for ammonia and NO_2^- production with Cu wafer and Cu foam(30s)@mesh. The electrolysis data were obtained after 30 min of potentiostatic electrolysis at pH 14 in 100 mmol L^{-1} KNO_3 electrolyte solution. For each electrolysis a newly prepared catalyst was used. The table displays the averaged FE values from three individual electrolyses per applied potential. The presented data correspond to **Figure S20**.

Table S16. Literature survey: Ammonia production in terms of FE and PCD at different potentials through electrochemical NO_3RR, utilizing H-type cells with and **without electrolyte stirring and flow cells.**

	Electrolyte	Condition	FE_{NH3} / %	E / V (vs. RHE)	PCD_{NH3} / $mA{\cdot}cm^2$	Ref.
Cu-foam(30 s)@mesh	0.5 M KNO_3+1 M KOH (pH 14)	H-type cell, 30min	75	-0.7	-1045	this work
Cu-foam(30 s)@mesh	0.5 M KNO_3 +1 M KOH (pH 14)	H-type cell, 30min	95	-0.5	-710	this work
Cu-foam(30 s)@mesh	0.5 M KNO_3 +1 M KOH (pH 14)	H-type cell, 30min	85	-0.3	-420	this work
Cu-foam(30 s)@mesh	0.1M KNO_3 +1 M KOH (pH 14)	H-type cell, 30min	65	-0.7	-211	this work
Cu-foam(30 s)@mesh	0.5 M KNO_3 + 0.5 M K_2SO_4 (pH 7)	H-type cell, 30min	81	-1.0	-367	this work
$Cu_{50}Ni_{50}$/PTEF	0.1M KNO_3 +1M KOH (pH 14)	Gas-tight flow cell, 0.5mL/min	99.0	-0.15	-52.47	6
Cu/PTEF	0.1M KNO_3 + 1M KOH (pH 14)	Gas-tight flow cell, 0.5mL/min	99	-0.20	-41.2	6
Cu50Ni50	1M KOH + 0.1M KNO3(RDE,400rpm	Gas-tight flow cell, 0.5mL/min	~100	-0.1	-90	6
		Gas-tight flow cell, 0.5mL/min,	~100	-0.2	-300	6
Cu/Cu_2O NWAs/Cu mesh	14 mM KNO_3 + 0.5M Na_2SO_4 (pH 7)	H-type cell, 2h, stirring with 300 rpm	95.8	-0.85	-38.32	7
Cu NC	0.1M KNO_3 + 1M KOH (pH 14)	H-type cell, 30min	84.7	-1.0	-39.8	8
Ru nano crystal	0.1M KNO_3 + 1 M KOH (pH 14)	Gastight two-compartment H-cell, Ar 10sccm, 1h, stirring with 300 rpm	100	-0.2	-25.0	9
Ru nano crystal	1 M KNO_3 + 1 M KOH (pH 14)	Gastight two-compartment H-cell, Ar 10sccm, 1h, stirring with 300 rpm	100	-0.2	-125.0	9
5nm Fe/CFP	0.4M KNO_3 + 1 M Phosphate buffer (pH 7)	Air tight H-type cell, 1h, with Ar bubbling 20 sccm	83	-0.85	-30	10
Ultra-thin CoOx	0.1M KNO_3 + 1 M KOH (pH 14)	H-type cell, 1h	93.4	-0.3	-2.9	11
Rh NC	0.1M KNO_3 + 0.5 M Na_2SO_4 (pH 7)	H-type cell, 3min, stirring with 500rpm	75	-0.2	-126	12
CuPd aerogel	50ppm KNO_3 + 0.5M K_2SO_4 (pH 7)	H-type cell, 2h stirring with 500rpm	83.4	-0.46	-13.84	13
Fe Single atom	0.5M KNO_3 + 0.1M K_2SO_4 (pH 7)	H-type cell, 2h stirring with 500rpm	75	-0.46	-37.9	14
Au with surface adsorbed thiourea	0.5 M $NaNO_3$ (pH 12.5)	H-type cell, H_2 bubbled, 6h	85	-0.5	-2.85	15
Ti electrode	0.3M KNO_3 + 0.1 M HNO_3	Compression cell, bubble rate using 20 sccm, 30min	82	-1.0	-22	16
$NiFe_2O_4$ nano sheet	0.1M $NaNO_3$ + 0.1 M Phosphate buffer (pH 7)	H-type cell, 1h	97	-0.6	-48.3	17
Cu-PTCDA	500 ppm NO_3^- + 0.1 M Phosphate buffer (pH 7)	H-type cell, 1h	77	-0.4	-15	18

TiO_{2-x36}	3.6 mM $NaNO_3$ + 0.5 M Na_2SO_4 (pH 7)	H-type cell, 2h, stirring with 500 rpm	85	-1.0	-12	19
Cu-BTC-MOF	1M KOH + 0.1M KNO_3	H-type cell, Ar gas was continuously pumped	95	-0.2V	-261	20
MP-Cu	1M KOH + 0.05M KNO_3	H-type cell, 1.5h, stirring with 500 rpm	100	-0,3	-135	21
CuO NWA@Co3O4	1M KOH + 1,400 ppm NO_3^-	H-type cell, 2.5h, stirring with 300 rpm	96	-0,23	-441	22
Cu0.86Ir0.14OzNS	1M KOH + 0.05M KNO_3	H-type cell, 4h	92 @ 4hrs		-100	23
Ru-CuNW	1M KOH + 2,000 ppm NO_3	Flow-system H-cell, 10min, stirring with 1600 rpm	95.6	-0.1	-963	24
CuCoSP	0.1M KOH + 0.1M KNO_3	H-type cell, 1 h, stirring with 1600 rpm	90.6	-0.175	-227.8	25
Cu-NBs-100	1M KOH + 0.1M KNO_3	H-type cell, continuously purging with Ar	95	0	-190	26
			95.3	-0.15	-273.6	
			93.3	-0.25	-371.3	
Co-NA's	1M KOH + 0.1M KNO_3	H-type cell, 200s, stirring with 400 rpm	~100	-0.09	-500	27
	1M KOH + 0.1M KNO_3		~100	-0.24	-2200	
	0.5M Na_2SO_4 + 0.1M KNO_3		~96	-0.4	-240	
	0.5M Na_2SO_4 + 0.1M KNO_3		~96	-0.6	-652.8	
Cu50Co50/Ni Foam	1M KOH + 0.1M KNO_3	H-type cell, 600s, stirring with 1000 rpm	88	0	-347.6	28
			100	-0.2	-1035	
			90	-0.4	-990	

References

(1) Vanýsek, P. Ionic conductivity and diffusion at infinite dilution, in Haynes, W. M. (Ed.) *CRC Handbook of Chemistry and Physics*, 97th Ed., **2016**, CRC Press, Boca Raton, pp. 5-76.

(2) Ibl, N.; Kind, R.; Adam, E. Mass transfer at electrodes with gas stirring, *An. Quím.* **1975**, 71, 1008–1016.

(3) Venczel, J. Über den Stofftransport an gasentwickelnden Elektroden, PhD thesis, *Eidgenössische Technische Hochschule Zürich*, **1961**.

(4) Ibl, N.; Venczel, J. Untersuchung des Stofftransports an gasentwickelnden Elektroden, *Metalloberfläche* **1970**, 24, 365–374.

(5) Sides, P. J. Phenomena and effects of electrolytic gas evolution, in White, R. E.; Bockris, J. O'M.; Conway, B. E. (Eds.) *Modern Aspects of Electrochemistry*, **1986**, Plenum Press, New York, vol. 8, pp. 303–354

(6) Wang, Y.; Xu, A.; Wang, Z.; Huang, L.; Li, J.; Li, F.; Wicks, J.; Luo, M.; Nam, D.-H.; Tan, C.-S.; Ding, Y.; Wu, J.; Lum, Y.; Dinh, C.-T.; Sinton, D.; Zheng, G.; Sargent, E. H. Enhanced Nitrate-to-Ammonia Activity on Copper–Nickel Alloys via Tuning of Intermediate Adsorption. *J. Am. Chem. Soc.* **2020,** 142, 5702-5708.

(7) Wang, Y.; Zhou, W.; Jia, R.; Yu, Y.; Zhang, B. Unveiling the Activity Origin of a Copper-based Electrocatalyst for Selective Nitrate Reduction to Ammonia. *Angew. Chem., Int.* **2020,** 59, 5350-5354.

(8) Yang, J.; Qi, H.; Li, A.; Liu, X.; Yang, X.; Zhang, S.; Zhao, Q.; Jiang, Q.; Su, Y.; Zhang, L.; Li, J.-F.; Tian, Z.-Q.; Liu, W.; Wang, A.; Zhang, T. Potential-Driven Restructuring of Cu Single Atoms to Nanoparticles for Boosting the Electrochemical Reduction of Nitrate to Ammonia. *J. Am. Chem. Soc.* **2022**, 144, 12062-12071.

(9) Li, J.; Zhan, G.; Yang, J.; Quan, F.; Mao, C.; Liu, Y.; Wang, B.; Lei, F.; Li, L.; Chan, A. W. M.; Xu, L.; Shi, Y.; Du, Y.; Hao, W.; Wong, P. K.; Wang, J.; Dou, S.-X.; Zhang, L.; Yu, J. C. Efficient Ammonia Electrosynthesis from Nitrate on Strained Ruthenium Nanoclusters. *J. Am. Chem. Soc.* **2020**, 142, 7036-7046.

(10) Harmon, N. J.; Rooney, C. L.; Tao, Z.; Shang, B.; Raychaudhuri, N.; Choi, C.; Li, H.; Wang, H. Intrinsic Catalytic Activity of Carbon Nanotubes for Electrochemical Nitrate Reduction. *ACS Catal.* **2022**, 12, 9135-9142.

(11) Wang, J.; Cai, C.; Wang, Y.; Yang, X.; Wu, D.; Zhu, Y.; Li, M.; Gu, M.; Shao, M. Electrocatalytic Reduction of Nitrate to Ammonia on Low-Cost Ultrathin CoOx Nanosheets. *ACS Catal.* **2021**, 11, 15135-15140.

(12) Liu, H.; Lang, X.; Zhu, C.; Timoshenko, J.; Rüscher, M.; Bai, L.; Guijarro, N.; Yin, H.; Peng, Y.; Li, J.; Liu, Z.; Wang, W.; Cuenya, B. R.; Luo, J. Efficient Electrochemical Nitrate Reduction to Ammonia with Copper-Supported Rhodium Cluster and Single-Atom Catalysts. *Angew. Chem., Int.* **2022**, 61, e202202556.

(13) Xu, Y.; Ren, K.; Ren, T.; Wang, M.; Liu, M.; Wang, Z.; Li, X.; Wang, L.; Wang, H. Cooperativity of Cu and Pd active sites in CuPd aerogels enhances nitrate electroreduction to ammonia. *Chem. Commun.* **2021**, 57, 7525-7528.

(14) Wu, Z.-Y.; Karamad, M.; Yong, X.; Huang, Q.; Cullen, D. A.; Zhu, P.; Xia, C.; Xiao, Q.; Shakouri, M.; Chen, F.-Y.; Kim, J. Y.; Xia, Y.; Heck, K.; Hu, Y.; Wong, M. S.; Li, Q.; Gates, I.; Siahrostami, S.; Wang, H. Electrochemical ammonia synthesis via nitrate reduction on Fe single atom catalyst. *Nat. Commun.* **2021**, 12, 2870.

(15) El-Deab, M. S. Electrochemical reduction of nitrate to ammonia at modified gold electrodes. *Electrochim. Acta* **2004**, 49, 1639-1645.

(16) McEnaney, J. M.; Blair, S. J.; Nielander, A. C.; Schwalbe, J. A.; Koshy, D. M.; Cargnello, M.; Jaramillo, T. F. Electrolyte Engineering for Efficient Electrochemical Nitrate Reduction to Ammonia on a Titanium Electrode. *ACS Sustain. Chem. Eng.* **2020**, 8, 2672-2681.

(17) Xie, L.; Hu, L.; Liu, Q.; Sun, S.; Zhang, L.; Zhao, D.; Liu, Q.; Chen, J.; Li, J.; Ouyang, L.; Alshehri, A. A.; Kong, Q.; Sun, X. High-performance electrochemical nitrate reduction to ammonia under ambient conditions using $NiFe_2O_4$ nanosheet arrays. *Inorg. Chem. Front.* **2022**, 9, 3392-3397.

(18) Chen, G.-F.; Yuan, Y.; Jiang, H.; Ren, S.-Y.; Ding, L.-X.; Ma, L.; Wu, T.; Lu, J.; Wang, H. Electrochemical reduction of nitrate to ammonia via direct eight-electron transfer using a copper–molecular solid catalyst. *Nat. Energy* **2020**, 5, 605-613.

(19) Jia, R.; Wang, Y.; Wang, C.; Ling, Y.; Yu, Y.; Zhang, B. Boosting Selective Nitrate Electroreduction to Ammonium by Constructing Oxygen Vacancies in TiO_2. *ACS Catal.* **2020**, 10, 3533-3540.

(20) Yu, J.; Qin, Y.; Wang, X.; Zheng, H.; Gao, K.; Yang, H.; Xie L.; Hu Q.; He C. Boosting electrochemical nitrate-ammonia conversion via organic ligands-tuned proton transfer. *Nano Energy* **2022,** 103, 107705.

(21) Wen, W.; Yan, P.; Sun, W.; Zhou, Y.; Yu, X. Metastable Phase Cu with Optimized Local Electronic State for Efficient Electrocatalytic Production of Ammonia from Nitrate. *Adv. Funct. Mater.* **2023**, 33(6), 2212236

(22) Liu, H.; Li, J.; Du, F.; Yang, L.; Huang, S.; Gao, J.; Li C.; Guo, C. A core–shell copper oxides-cobalt oxides heterostructure nanowire arrays for nitrate reduction to ammonia with high yield rate. Green Energy Environ. **2022**-in press, https://doi.org/10.1016/j.gee.2022.03.00.

(23) Akram, M. A.; Zhu, B.; Cai, J.; Qin, S.; Hou, X.; Jin, P.; Wang, F.; He, Y.; Li, X.; Feng, L. Hierarchical Nanospheres with Polycrystalline Ir&Cu and Amorphous Cu_2O toward Energy ‐ Efficient Nitrate Electrolysis to Ammonia. *Small*, **2023**, 2206966.

(24) Chen, F. Y.; Wu, Z. Y.; Gupta, S.; Rivera, D. J.; Lambeets, S. V.; Pecaut, S.; Kim, J.; Zhu, P.; Finfrock Y.; Meira, D.M.; King, G.; Gao, G.; Xu, W.; Cullen, D.A.; Zhou, H.; Han, Y.; Perea, D.E.;Muhich, C.L.; Wang, H. Efficient conversion of low-concentration nitrate sources into ammonia on a Ru-dispersed Cu nanowire electrocatalyst. *Nat. Nanotechnol.*, **2022**,17(7), 759-767.

(25) He, W.; Zhang, J.; Dieckhöfer, S.; Varhade, S.; Brix, A. C.; Lielpetere, A., Seisel, S.; Junqueria J.R.C.; Schuhmann, W. Splicing the active phases of copper/cobalt-based catalysts achieves high-rate tandem electroreduction of nitrate to ammonia. *Nat. Commun.*, **2022**,13(1), 1129.
(26) Hu, Q.; Qin, Y.; Wang, X.; Wang, Z.; Huang, X.; Zheng, H.; Gao,K.;Yang, H.;Zhang,P.;Shao,M.; He, C. Reaction intermediate-mediated electrocatalyst synthesis favors specified facet and defect exposure for efficient nitrate–ammonia conversion. Energy Environ. Sci., **2021**,14(9), 4989-4997.
(27) Deng, X.; Yang, Y.; Wang, L.; Fu, X. Z.; & Luo, J. L.. Metallic Co nanoarray catalyzes selective NH_3 production from electrochemical nitrate reduction at current densities exceeding 2 A cm^{-2}. *Adv. Sci.*, **2021**,8(7), 2004523.
(28) Fang, J. Y.; Zheng, Q. Z.; Lou, Y. Y.; Zhao, K. M.; Hu, S. N.; Li, G., Akdim,O.;Huang,X.; Sun, S. G. Ampere-level current density ammonia electrochemical synthesis using CuCo nanosheets simulating nitrite reductase bifunctional nature. *Nat. Commun.*, **2022**,13(1), 7899.
